# Carpel size, grain filling, and morphology determine individual grain weight in wheat

**DOI:** 10.1093/jxb/erv378

**Published:** 2015-08-05

**Authors:** Quan Xie, Sean Mayes, Debbie L. Sparkes

**Affiliations:** Division of Plant and Crop Sciences, The University of Nottingham, Sutton Bonington Campus, Loughborough, Leicestershire LE12 5RD, UK

**Keywords:** Carpel, distal grain, grain filling, grain morphology, grain water, grain weight, QTL, spelt, wheat.

## Abstract

Individual grain weight is a major yield component in wheat. To provide a comprehensive understanding of grain weight determination, the carpel size at anthesis, grain dry matter accumulation, grain water uptake and loss, grain morphological expansion, and final grain weight at different positions within spikelets were investigated in a recombinant inbred line mapping population of bread wheat (*Triticum aestivum* L.)×spelt (*Triticum spelta* L.). Carpel size, grain dry matter and water accumulation, and grain dimensions interacted strongly with each other. Furthermore, larger carpels, a faster grain filling rate, earlier and longer grain filling, more grain water, faster grain water absorption and loss rates, and larger grain dimensions were associated with higher grain weight. Frequent quantitative trait locus (QTL) coincidences between these traits were observed, particularly those on chromosomes 2A, 3B, 4A, 5A, 5DL, and 7B, each of which harboured 16−49 QTLs associated with >12 traits. Analysis of the allelic effects of coincident QTLs confirmed their physiological relationships, indicating that the complex but orderly grain filling processes result mainly from pleiotropy or the tight linkages of functionally related genes. After grain filling, distal grains within spikelets were smaller than basal grains, primarily due to later grain filling and a slower initial grain filling rate, followed by synchronous maturation among different grains. Distal grain weight was improved by increased assimilate availability from anthesis. These findings provide deeper insight into grain weight determination in wheat, and the high level of QTL coincidences allows simultaneous improvement of multiple grain filling traits in breeding.

## Highlight

A high level of QTL coincidences was found for the traits that determine individual grain weight in wheat: carpel size, grain dry matter and water accumulation, and grain morphology.

## Introduction

Individual grain weight is a major yield determinant in wheat, and therefore a key breeding target to boost yield for global food security. In addition, larger grains are also preferred for their better milling performance and end-use quality (Gegas *et al.*, 2010). Grain weight is mainly determined through the grain filling between anthesis and maturity, during which there are three physiological processes occurring simultaneously: grain dry matter accumulation, grain water accumulation and subsequent desiccation, and grain morphological expansion.

Grain dry matter accumulation is a process of deposition of starch (~60–70% of the mature grain weight), proteins (8–15%), and other nutrients (e.g. minerals, vitamins, and fibres) (Shewry, 2009). The assimilates for grain filling originate primarily from current photosynthesis and the remobilization of soluble reserves accumulated in the vegetative organs before anthesis. Senescing organs can also supply some assimilates by transporting the nutrients from the structural macromolecule degradation into developing grains at the late stage of plant growth (Distelfeld *et al.*, 2014). There is ample evidence that combined current photosynthetic capacity and reserve remobilization are in excess of the demand of the growing grains; that is, grains are mainly sink limited after anthesis (Slafer and Savin, 1994; Borras *et al.*, 2004; Miralles and Slafer, 2007). During the post-anthesis period, therefore, the factors limiting synthesis and deposition of storage products within grains need to be determined. Grain filling can be divided into two components: rate and duration. Grain filling rate follows a slow–fast–slow pattern (Shewry *et al.*, 2012), reflecting the biochemical reaction efficiency for starch and protein synthesis (Shewry *et al.*, 2009). In contrast, its duration reflects the timing of the grain filling progress. The rate and duration of grain filling both contribute to final grain weight; however, there is occasionally a negative relationship between these two components (Charmet *et al.*, 2005; Wang *et al.*, 2009; Gonzalez *et al.*, 2014). Despite the importance of grain dry matter accumulation, only a few studies have been conducted to determine its genetic basis, including gene expression analysis (Laudencia-Chingcuanco *et al.*, 2007; Gillies *et al.*, 2012) and quantitative trait locus (QTL) identification (Charmet *et al.*, 2005; Wang *et al.*, 2009).

The dynamics of grain water accumulation appears to be ‘bell’ shaped: water is absorbed rapidly until a plateau is reached, and then lost quickly during grain desiccation (Barlow *et al.*, 1980; Lizana *et al.*, 2010). Water is essential to transport photoassimilates and other nutrients into developing grains. It also provides a suitable environment for metabolic processes, and directly takes part in the synthesis of storage products. A strong association between maximum grain water content and final grain weight has been found in wheat (Lizana *et al.*, 2010; Hasan *et al.*, 2011; Gonzalez *et al.*, 2014), and in other crops such as maize (*Zea mays* L.) (Borras *et al.*, 2003; Sala *et al.*, 2007) and sunflower (*Helianthus annuus* L.) (Rondanini *et al.*, 2009). However, little is known regarding the genetic determination of grain water dynamics.

Grain morphology changes along with dry matter and water accumulation. Immediately after fertilization, grain length, width, height (thickness), and thus volume increase rapidly. The first dimension to reach its maximum value is grain length (~15 d after anthesis), followed by grain width, height, and volume (~28 d) (Lizana *et al.*, 2010; Hasan *et al.*, 2011), corresponding to the period of endosperm cell enlargement (Briarty *et al.*, 1979). Expansins, a type of protein inducing cell wall extension, have been found to be associated with grain size dynamics (Lizana *et al.*, 2010). Grain dimensions then decrease slightly, and reach their final size at maturity (Millet and Pinthus, 1984; Lizana *et al.*, 2010). Final grain length, width, height, and volume are closely associated with grain weight (Millet and Pinthus, 1984; Breseghello and Sorrells, 2007; Gegas *et al.*, 2010; Lizana *et al.*, 2010; Hasan *et al.*, 2011), and many QTLs for these traits have been identified (Breseghello and Sorrells, 2007; Gegas *et al.*, 2010; Williams *et al.*, 2013).

There is a great variation in final grain weight within spikes for a given genotype. A spike of wheat comprises ~16–25 spikelets, and each spikelet sets ~0–5 grains, depending on genotype, environment, and spikelet position within the spike. The second grain (G2) from the rachis is usually largest, followed by the first (G1), third (G3), and more distal ones, if present (Calderini and Reynolds, 2000; Calderini and Ortiz-Monasterio, 2003; Hasan *et al.*, 2011). The average grain weight across different positions within spikelets would be increased by ~15% (estimated from numerous previous studies) if all other grains reach the grain weight of G2. For this to be realized, the distal grains (G3 and farther) therefore need to be enlarged. It has been observed that the distal florets produce smaller carpels at anthesis (Singh and Jenner, 1982; Calderini *et al.*, 1999). During grain filling, distal grains show a slower rate and shorter effective duration of grain filling (Simmons and Crookston, 1979; Millet and Pinthus, 1984), lower grain water content, and smaller grain dimensions (Lizana *et al.*, 2010; Hasan *et al.*, 2011), probably as a consequence of having fewer endosperm cells (Singh and Jenner, 1982; Gao *et al.*, 1992).

How grain weight is determined throughout the grain filling has only been partially elucidated in wheat. Earlier studies usually focused on part of the grain filling processes through experiments evaluating a few contrasting genotypes. In this study, a large number of genotypes from a bread wheat×spelt mapping population were used, and a wide range of key traits (carpel size at anthesis, grain dry matter accumulation, grain water uptake and loss, grain morphological expansion, and final grain weight) were analysed to provide a comprehensive understanding of grain weight determination. Subsequently, the genetic loci underlying these traits were identified. To understand the grain weight variation within spikelets, the physiological and genetic differences in grain filling patterns between distal and basal grains were then evaluated in detail.

## Materials and methods

### Plant materials and field experiments

A mapping population of Swiss winter bread wheat (*T. aestivum* L. ‘Forno’)×Swiss winter spelt (*T. spelta* L. ‘Oberkulmer’), consisting of 226 F_5_ recombinant inbred lines (RILs) (Messmer *et al.*, 1999), was used in this study. All the RILs, together with two parents and another bread wheat *T. aestivum* L. ‘Duxford’, were grown at University of Nottingham Farms, Leicestershire, UK in two growing seasons: 2011–2012 and 2012–2013. Field experiments were based on a randomized complete block design with three replicates in each season. Seeds were sown at 250 seeds m^–2^, with plot size 6×1.6 m in 2011–2012 and 12×1.6 m in 2012–2013. The soil was a sandy loam, with pH 7.6, and contained 78.2kg and 70.4kg nitrogen ha^–1^ in the top 90cm profile in 2012 and 2013, respectively. Fertilizers (nitrogen, potassium, and phosphorus) were applied according to standard recommended agronomic practice. A prophylactic programme of disease, weed, and pest management was used to maintain undisturbed healthy crop growth. To avoid the confounding effects of different crop developmental stages on grain filling traits, a subset consisting of 72 RILs was selected in 2012, based on similar flowering dates in 2010 (±4 d) and 2011 (±1 d). This subset was enlarged to 110 RILs in 2013. Phenotypic measurements were carried out in the subsets, with the exception of final grain dimensions in 2013, which were measured on 226 RILs.

### Carpel dissection at anthesis

**Fig. 1.**
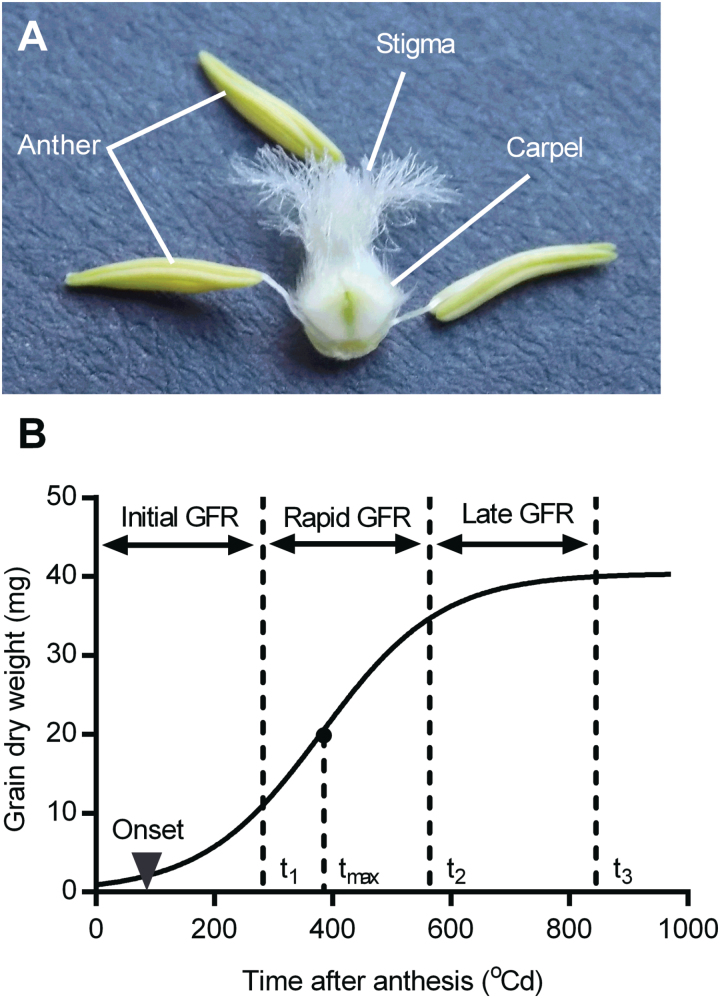
Carpel from a wheat floret (A) and a schematic diagram of grain dry matter accumulation (B). Data of grain dry weight were fitted to a logistic growth curve over time after anthesis. Total grain filling duration (*t*_3_) was divided equally into three phases: initial (anthesis to *t*_1_), rapid (*t*_1_ to *t*_2_), and late (*t*_2_ to *t*_3_). The average grain filling rate (GFR) during each phase was calculated, and termed the initial, rapid, and late GFR. Onset of grain filling and maximum grain filling rate are indicated by a triangle and filled circle (at the time *t*_max_), respectively. (This figure is available in colour at *JXB* online.)

The carpel is the unfertilized female organ, containing ovary, style, and feathery stigma (Fig. 1A). Five main spikes from each plot of the subsets were sampled when the first anthers in the middle of the spikes were just visible. In 2012, the two middle spikelets of each spike were collected. To represent a spike better, in 2013 three different spikelets along one side of each spike were selected: the third from the bottom, the third from the top, and the middle one between them. Carpels from the first, second, and third florets within spikelets counting from the rachis, namely Carpel 1 (C1), Carpel 2 (C2), and Carpel 3 (C3), were removed carefully using forceps. The fourth and more distal florets were discarded as they finally became infertile in most RILs. C1, C2, and C3 across all spikelets sampled from the five spikes were pooled, respectively, and dried in an oven at 85 °C for 48h. Carpel dry weight was recorded using an electronic balance (±0.0001g) (125A, Precisa, Switzerland).

### Grain dry matter and water accumulation

From anthesis onwards, the dynamics of grain dry matter and water accumulation were investigated until maturity. Two representative spikes (but five spikes at anthesis and maturity) from each plot were collected every 5 d. Spikelet sampling and dissecting followed the same procedure as for carpel analysis. Grains from the first, second, and third florets within spikelets counting from the rachis were named Grain 1 (G1), Grain 2 (G2), and Grain 3 (G3), respectively. G1, G2, and G3 were weighed immediately for fresh weight and again after drying at 85 °C for 48h, and grain water content was calculated as the difference between them.

For each of G1, G2, and G3, a logistic growth curve was fitted to the grain dry weight data (Fig. 1B) (Zahedi and Jenner, 2003; Wang *et al.*, 2009).

$$W_{\text{d}}=A+\frac{C}{1+{\text{e}}^{-B(t-M)}}$$

where *W*_d_ is the individual grain dry weight, *A* is the lower asymptote, (*A*+*C*) is the upper asymptote (final grain weight), *B* is the doubled relative growth rate at the time *M*, *M* is the time when the absolute grain filling rate is at maximum, and when grains grow to (*A*+0.5*C*), and *t* is the accumulated thermal time after anthesis in degree days (°Cd; degree days after anthesis, DAA).

Grain filling duration (*t*_3_) was calculated from anthesis to the time when grains had grown to (*A*+0.99*C*) (*t*_3_=*M*+4.5951/*B*). This period was then divided equally into three phases, corresponding to the time courses of endosperm cell division and grain expansion, rapid grain filling, and maturation, respectively (Shewry *et al.*, 2012). The grain filling rates during each phase and across the whole grain filling period were calculated, and termed initial, rapid, late, and average grain filling rates. Onset of grain filling (*t*_onset_) was calculated when grains had grown to (*A*+0.05*C*) (*t*_onset_=*M*–2.9444/*B*). At the time *M* (*t*_max_), the maximum grain filling rate (MGFR) was reached (MGFR=*BC*/4).

For the water content of G1, G2, and G3, a cubic function was fitted.

$$W_{\text{w}}=b_{\text{3}}t^{\text{3}}+b_{\text{2}}t^{\text{2}}+b_{\text{1}}t+a$$

where *W*_w_ is the individual grain water content, *t* is the accumulated thermal time after anthesis, and *b*_3_, *b*_2_, *b*_1_, and *a* are coefficients.

When d*W*_w_/d*t*=0, *W*_w_=*W*_max_ (maximum water content, MWC), *t=t*_mwc_ (the time at maximum water content),

$$W_{\text{max}}=b_{3}t_{\text{mwc}}^{3}+b_{2}t_{\text{mwc}}^{2}+b_{1}t_{\text{mwc}}+a$$

$$t_{\text{mwc}}=\frac{-b_{2}-\sqrt{b_{2}^{2}-3b_{1}b_{3}}}{3b_{3}}$$

Average water absorption rate and water loss rate (desiccation) of grains were also calculated as the slopes of linear functions from anthesis to *t*_mwc_, and from *t*_mwc_ to the time for last measurement, respectively.

### Grain dimensions

Ten genotypes differing in grain weight were selected to observe the dynamics of grain expansion in 2013. Grain samples were the same as those used for grain dry matter and water analysis. After dissection, grain length, width, and height (thickness, grain crease downward) of G1, G2, and G3 were measured immediately using an electronic calliper (OD-15CP, Mitutoyo, UK).

At maturity, grain dimensions across G1, G2, and G3 were evaluated in the subset and 226 RILs in 2012 and 2013, respectively. In 2012, five spikes from a plot were sampled, and each of them was divided from the bottom to the top equally into three parts; all spikelets from each part were then dissected as G1, G2, and G3 (nine grain groups in total). One representative grain from each group (i.e. nine grains for each plot) was measured for grain length, width, and height using an electronic calliper (OD-15CP). In 2013, digital image analysis for grain dimensions was used (Breseghello and Sorrells, 2007). For each plot, 20 grains were extracted randomly from the combined samples, and spread onto a scanner bed (Officejet 4500, HP, USA). For each sample, two images, one with grain crease downward and the other with lateral side downward, were taken at a resolution of 200 ppi. With the software ImageJ (National Institutes of Health, USA, http://rsbweb.nih.gov/ij/), the images were segmented into intact grains and background using the ‘Color Threshold’ feature, and grain dimensions were measured using the ‘Analyze Particles’ feature by fitting the best ellipses. Major and minor axes of the best fitting ellipses corresponded to grain length and width (the first image) or grain length and height (the second image), respectively.

Grain volume was calculated by considering grain as an ellipsoid and applying the geometric formula: *V*_g_=(4/3)*πabc*, where *V*_g_ is the grain volume, *π*=3.1416, *a*=0.5 grain length, *b*=0.5 grain width, and *c*=0.5 grain height (Breseghello and Sorrells, 2007; Hasan *et al.*, 2011). In addition, the ratios of grain length to width (L/W) and of grain length to height (L/H) were calculated as grain shape parameters (Breseghello and Sorrells, 2007; Gegas *et al.*, 2010).

### Timing of rapid flag leaf senescence

Ten genotypes, as described previously, were used to determine the timing of rapid flag leaf senescence. In 2012, a scale of 10 (fully green) to 0 (fully senesced) was used for visual assessment based on the whole canopy. Assessment started from anthesis at 5 d intervals until maturity. In 2013, flag leaves from the same shoots used for grain dimension analysis were collected at 5 d intervals. Green and yellow parts of each leaf were separated, and both measured for area using an area meter (LI3100, LI-COR, USA). The percentage of green area was used to quantify the senescence progress.

A Gompertz descending curve was then fitted to the data of visual scoring and percentage green area (Gooding *et al.*, 2000).

$$G=A+C{\text{e}}^{-{\text{e}}^{-B\left(t-M\right)}}$$

where *G* is the visual scores or percentage green area, *A* is the lower asymptote (fully senesced), (*A+C*) is the upper asymptote (the initial values), *B* is the relative senescence rate at the time *M*, *M* is the time when the maximum senescence rate (MSR) is reached (*t*_msr_), and *t* is the accumulated thermal time after anthesis.

### De-graining at anthesis

A de-graining experiment was conducted in two bread wheat cultivars Forno and Duxford in 2013. Five main spikes from a plot were selected at anthesis, and all the spikelets along one side of each spike removed (virtually doubling the assimilate availability for the remaining grains), while five intact spikes were used as control. G1, G2, and G3 from the de-graining and control spikes were recorded for dry weight at maturity.

### Statistical analysis

Analysis of variance and multiple comparisons (Fisher’s unprotected least significant difference) were performed to test the phenotypic differences in grain filling traits among genotypes and among G1, G2, and G3. Pearson correlation and regression analyses were used to determine the relationships between different traits. Broad sense heritability (*H* ^2^) of each trait across years was calculated as: $H^{2}\;=\;\sigma_{g}^{2}/(\sigma_{g}^{2}\;+\;\sigma_{ge}^{2}/n\;+\;\sigma_{e}^{2}/rn)$, where $\sigma_{g}^{2}$ is the genotypic variance, $\sigma_{ge}^{2}$ is the genotype-by-environment interaction variance, $\sigma_{e}^{2}$ is the error variance, *n* is the environment number (years), and *r* is the replicate number per environment (Borràs-Gelonch *et al.*, 2012). To estimate the variance components, a linear mixed model, using the method of residual maximum likelihood (REML), was used. The environment (year) and replicate were set as fixed factors, and genotype and genotype-by-environment interaction as random factors. All statistical analyses, including curve fitting, were performed using Genstat v17 and GraphPad Prism v6.05.

### QTL analysis

A total of 182 molecular markers (restriction fragment length polymorphisms and simple sequence repeats), resulting in 230 segregating loci, were used to establish the genetic map of Forno×Oberkulmer (Messmer *et al.*, 1999). Linkage analysis was performed with the package JoinMap v4 (Van Ooijen, 2006), and 23 linkage groups were produced, covering 2469 cM with an average marker density of 13.6 cM. QTL analysis was performed with the package MapQTL v6 (Van Ooijen, 2009), using the mean values over replicates of phenotypic data for each environment. Interval mapping was carried out to estimate the locations of significant QTLs, logarithm of the odds (LOD), additive effects, and the percentages of phenotypic variation explained by individual QTLs (*R* ^2^). Multiple-QTL model (MQM) mapping was then performed using the cofactors (the markers nearest to QTL peaks, tested for significance at *P*<0.02). A genome-wide significance threshold (*P*<0.05) was calculated for each trait through a permutation test with 1000 iterations. QTL nomenclature followed the Catalogue of Gene Symbols for Wheat (http://wheat.pw.usda.gov/GG2/Triticum/wgc/2008/). The alleles relatively increasing the values of the traits were defined as increasing alleles; otherwise as decreasing alleles. The linkage map and QTLs were drawn using the package MapChart v2.2 (Voorrips, 2002).

## Results

### Carpel size, grain dry matter and water accumulation, and grain dimensions are associated with final grain weight

Large differences between the parents and between the RILs in final grain weight and grain filling traits were found (Supplementary Table S1 available at *JXB* online), hence allowing further physiological and genetic analysis. *H* ^2^ of grain weight, average grain filling rate, grain water accumulation, and grain dimensions (except grain width) across different grain positions, were relatively high (0.68−0.81) (Supplementary Table S1), indicating strong genetic control.

**Fig. 2.**
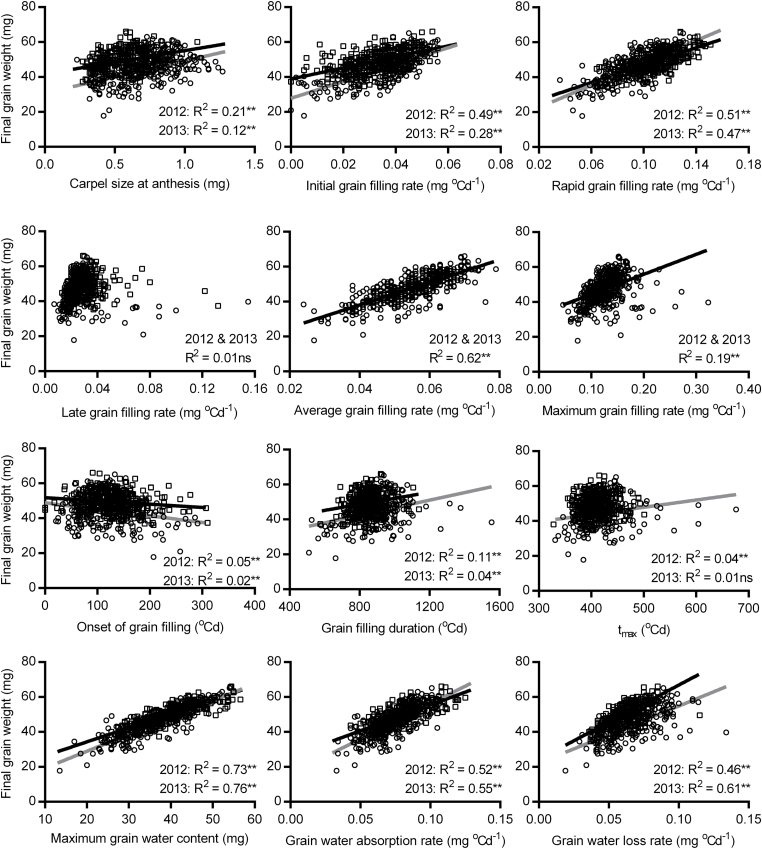
Relationships between carpel size at anthesis, grain dry matter and water accumulation, and final grain weight. Data in 2012 and 2013 are indicated by the circles (grey regression lines) and squares (black regression lines), respectively. Significance levels for regression analysis: ns, not significant; *P<0.05; **P<0.01. A common line is used to explain both years in the graphs indicated by ‘2012 & 2013’ if there are no significant differences in slopes and intercepts between two separate lines. Trait abbreviation: *t*_max_, time at the maximum grain filling rate.

Regression analysis showed that the carpel size at anthesis was positively associated with grain weight at maturity in both years (Fig. 2). Initial, rapid, and maximum grain filling rates were also positively associated with final grain weight, and there was a close relationship between average grain filling rate and final grain weight. In contrast, grain filling duration and *t*_max_ were positively but weakly associated with grain weight. The onset of grain filling, however, was negatively associated with grain weight, indicating the importance of earlier grain filling. In addition, grain water accumulation showed close relationships with final grain weight consistently across years, especially the maximum grain water content (Fig. 2).

**Fig. 3.**
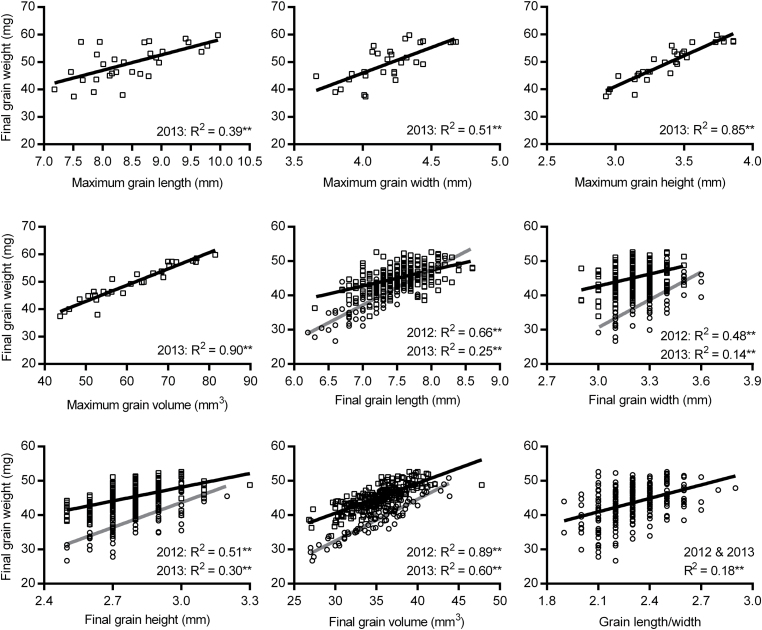
Relationships between grain dimensions and final grain weight. Data in 2012 and 2013 are indicated by the circles (grey regression lines) and squares (black regression lines), respectively. Significance levels for regression analysis: *P <0.05; **P< 0.01. A common line is used to explain both years in the graph indicated by ‘2012 & 2013’ if there are no significant differences in slopes and intercepts between two separate lines.

Grain dimensions expanded rapidly after anthesis (Supplementary Fig. S1 at *JXB* online), and grain length reached maximum first (410 DAA), followed by grain width, height, and volume (530 DAA). Grain length, width, height, and volume then decreased during the desiccation phase, by 6, 16, 13, and 30%, respectively. Maximum and final grain dimensions were positively associated with grain weight, in particular maximum grain height and volume (Fig. 3). Slimmer grains (L/W) appeared to be associated with slightly heavier grains.

### Carpel size, grain dry matter and water accumulation, and grain dimensions interact with each other

Larger carpels at anthesis greatly contributed to a higher initial rate, and, to a lesser extent, a rapid rate of grain filling; however, carpel size was negatively correlated with late grain filling rate (Table 1). Grain filling progress was also affected by carpel size: the larger carpels, the earlier grain filling and *t*_max_, and the slightly longer grain filling duration. In addition, carpel size was positively associated with maximum grain water content, grain water absorption, and loss rates (Table 1). Correlations between carpel size and maximum (and final) grain dimensions were also positive, although they were not significant.

**Table 1.** Phenotypic correlations of carpel size at anthesis with grain dry matter and water accumulation, and grain dimensions

Grain dry matter and water accumulation^*a*^	Carpel size at anthesis		Grain dimension	Carpel size at anthesis	
2012	2013	2012	2013
Initial grain filling rate	0.73**	0.51**	Maximum grain length	−^b^	0.19
Rapid grain filling rate	0.21**	0.14**	Maximum grain width	−	0.25
Late grain filling rate	–0.31**	–0.26**	Maximum grain height	−	0.24
Average grain filling rate	0.28**	0.20**	Maximum grain volume	−	0.30
Maximum grain filling rate	–0.10	–0.07	Final grain length	0.15	0.19
Onset of grain filling	–0.59**	–0.41**	Final grain width	–0.02	0.00
Grain filling duration	0.16*	0.11*	Final grain height	–0.08	0.28
*t*_max_	–0.25**	–0.32**	Final grain volume	0.01	0.24
Maximum grain water content	0.44**	0.33**	Grain length/width	0.17	0.16
Water absorption rate	0.49**	0.39**	Grain length/height	0.23	–0.08
Water loss rate	0.28**	0.16**			
*t*_mwc_	–0.14*	–0.24**			

^*a*^ Trait abbreviations: *t*_max_, time at the maximum grain filling rate; *t*_mwc_, time at the maximum grain water content.

^*b*^ Data absent.

*P<0.05, **P<0.01.

There were close relationships between grain water and dry matter accumulation (Table 2). Maximum grain water content, and grain water absorption and loss rates, strongly contributed to grain filling rates, especially the rapid one. A faster grain water absorption rate was associated with earlier grain filling and *t*_max_. Additionally, grain water accumulation strongly contributed to maximum and final grain dimensions, in particular grain height and volume (Table 2).

**Table 2.** Phenotypic correlations of grain water accumulation with grain dry matter accumulation and grain dimensions

Grain filling and dimension^*a*^	Maximum grain water content		Water absorption rate		Water loss rate		*t*_mwc_	
2012	2013	2012	2013	2012	2013	2012	2013
Initial grain filling rate	0.58**	0.44**	0.67**	0.50**	0.25**	0.26**	–0.26**	–0.30**
Rapid grain filling rate	0.84**	0.77**	0.69**	0.71**	0.75**	0.66**	0.28**	–0.04
Late grain filling rate	0.09	0.21**	–0.04	0.15**	0.33**	0.22**	0.34**	0.13*
Average grain filling rate	0.89**	0.87**	0.76**	0.82**	0.82**	0.72**	0.27**	–0.10
Maximum grain filling rate	0.51**	0.58**	0.34**	0.51**	0.64**	0.52**	0.39**	0.05
Onset of grain filling	–0.13	–0.07	–0.34**	–0.19**	0.21**	0.08	0.55**	0.37**
Grain filling duration	–0.09	–0.10	–0.08	–0.21**	–0.20**	0.02	0.00	0.31**
*t*_max_	–0.19**	–0.19**	–0.34**	–0.44**	–0.07	0.12*	0.42**	0.75**
Maximum grain length	−^b^	0.65**	−	0.57**	−	0.75**	−	0.09
Maximum grain width	−	0.78**	−	0.78**	−	0.52**	−	–0.36*
Maximum grain height	−	0.91**	−	0.86**	−	0.78**	−	–0.19
Maximum grain volume	−	0.95**	−	0.89**	−	0.85**	−	–0.15
Final grain length	0.65**	0.45*	0.52**	0.34	0.60**	0.54**	0.27*	0.25
Final grain width	0.40**	0.57**	0.21	0.58**	0.33**	0.29	0.39**	–0.33
Final grain height	0.69**	0.84**	0.59**	0.78**	0.47**	0.75**	0.20	–0.14
Final grain volume	0.76**	0.84**	0.59**	0.77**	0.60**	0.75**	0.34**	–0.07
Grain length/width	0.37**	0.09	0.38**	–0.01	0.36**	0.31	–0.02	0.39*
Grain length/height	–0.02	–0.28	–0.05	–0.34	0.13	–0.12	0.08	0.36*

^*a*^ Trait abbreviations: *t*_max_, time at the maximum grain filling rate; *t*_mwc_, time at the maximum grain water content.

^*b*^ Data absent.

*P<0.05, **P<0.01.

Maximum and final grain dimensions were strongly correlated (*r*=0.83−0.92, *P*<0.01). Both showed similar positive relationships with grain filling rates (Tables 3 and 4).

**Table 3.** Phenotypic correlations between maximum grain dimensions and grain dry weight accumulation

Grain filling trait	Maximum grain length	Maximum grain width	Maximum grain height	Maximum grain volume
Initial grain filling rate	0.16	0.67**	0.72**	0.62**
Rapid grain filling rate	0.75**	0.61**	0.76**	0.86**
Late grain filling rate	0.63**	0.12	0.30	0.46*
Average grain filling rate	0.71**	0.69**	0.85**	0.92**
Maximum grain filling rate	0.75**	0.50**	0.69**	0.80**
Onset of grain filling	0.25	–0.37*	–0.27	–0.13
Grain filling duration	–0.42*	–0.17	–0.17	–0.25
*t*_max_^*a*^	–0.15	–0.41*	–0.34	–0.31

^*a*^ *t*_max_, time at the maximum grain filling rate.

*P<0.05, **P<0.01.

**Table 4.** Phenotypic correlations between final grain dimensions and grain dry weight accumulation

Grain filling trait^*a*^	Grain length		Grain width		Grain height		Grain volume		Grain length/width		Grain length/height	
2012	2013	2012	2013	2012	2013	2012	2013	2012	2013	2012	2013
Initial GFR	0.42**	–0.05	0.26*	0.60**	0.39**	0.71**	0.45**	0.53**	0.24*	–0.34	0.04	–0.64**
Rapid GFR	0.60**	0.54**	0.35**	0.37*	0.54**	0.68**	0.65**	0.75**	0.34**	0.26	0.06	–0.07
Late GFR	0.49**	0.62**	0.26*	–0.03	0.45**	0.24	0.53**	0.43*	0.30**	0.53**	0.06	0.38*
Average GFR	0.64**	0.49**	0.38**	0.46**	0.59**	0.78**	0.71**	0.80**	0.37**	0.17	0.06	–0.20
Maximum GFR	0.59**	0.60**	0.34**	0.29	0.54**	0.61**	0.64**	0.72**	0.35**	0.35	0.06	0.05
Onset of GF	0.28*	0.40*	0.27*	–0.38*	0.26*	–0.28	0.35**	–0.06	0.07	0.53**	0.01	0.61**
GF duration	0.03	–0.02	0.20	0.12	–0.03	–0.07	0.05	–0.02	–0.12	–0.07	0.06	0.06
*t*_max_	0.19	0.29	0.37**	–0.19	0.12	–0.27	0.26*	–0.06	–0.09	0.34	0.08	0.50**

^*a*^ Trait abbreviations: GFR, grain filling rate; GF, grain filling; *t*_max_, time at the maximum grain filling rate.

*P<0.05, **P<0.01.

### QTL coincidences reflect the physiological relationships between grain filling traits and grain weight, and among grain filling traits

**Fig. 4.**
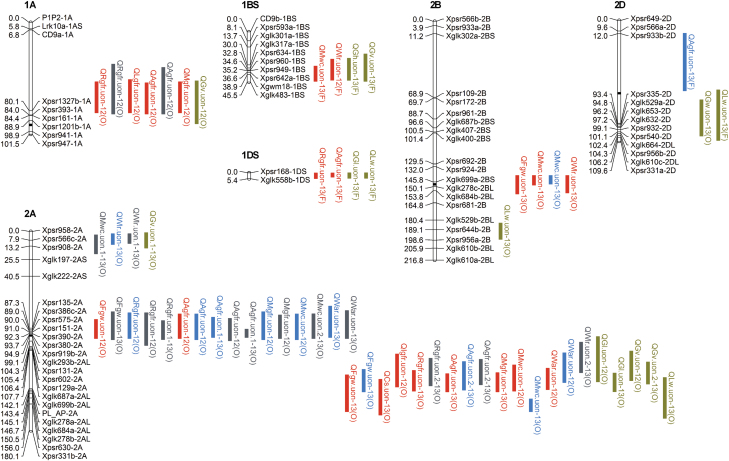

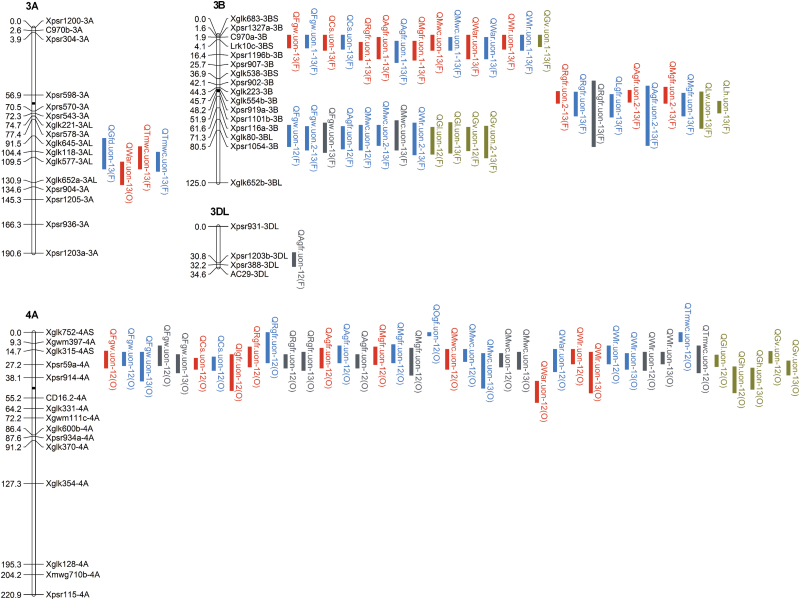

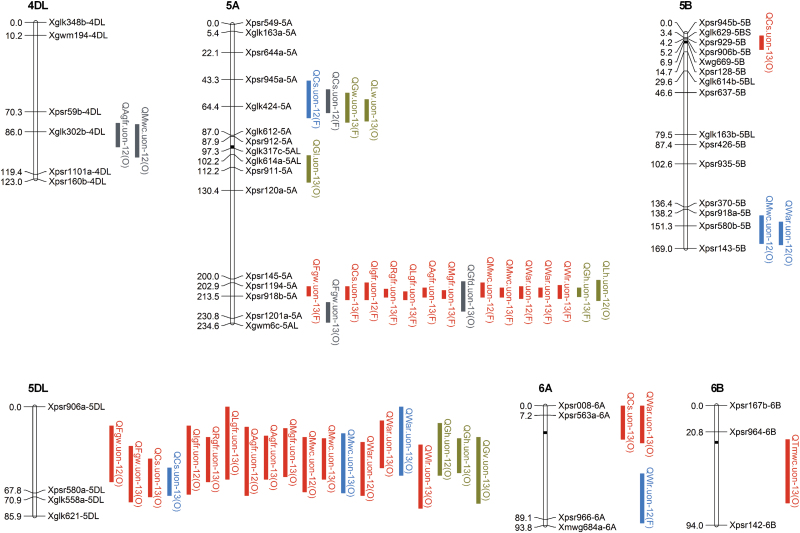

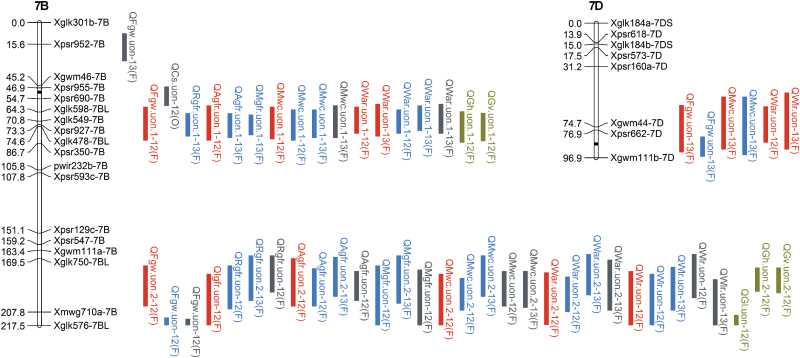
Quantitative trait locus (QTL) identification for grain weight, carpel size, grain dry matter and water accumulation, and grain dimensions. The 1–LOD support intervals of significant QTLs are indicated by red (Grain 1 or Carpel 1), blue (Grain 2 or Carpel 2), grey (Grain 3 or Carpel 3), and green (grain dimensions across different grain positions) vertical bars. A QTL symbol consists of a letter ‘Q’, trait abbreviation, laboratory name (*uon*), a serial number (if more than one QTL for the trait was detected on the same chromosome), a suffix 12 or 13 (QTL detected in 2012 or 2013), and the parentheses with the parent providing the increasing allele (increasing the value of the trait): F, bread wheat Forno; O, spelt Oberkulmer. Trait abbreviations: Fgw, final grain weight; Cs, carpel size; Igfr, initial grain filling rate; Rgfr, rapid grain filling rate; Lgfr, late grain filling rate; Agfr, average grain filling rate; Mgfr, maximum grain filling rate; Ogf, onset of grain filling; Gfd, grain filling duration; Tmax, time at tge maximum grain filling rate; Mwc, grain maximum water content; War, grain water absorption rate; Wlr, grain water loss rate; Tmwc, time at the maximum grain water content; Gl, grain length; Gw, grain width; Gh, grain height; Gv, grain volume; Lw, grain length/width; Lh, grain length/height.

A total of 249 significant QTLs were detected in the Forno×Oberkulmer mapping population across two years, comprising 26 QTLs for final grain weight, 13 for carpel size, 81 for grain dry matter accumulation, 90 for grain water accumulation, and 39 for final grain dimensions (Fig. 4; Supplementary Table S2 at *JXB* online). These QTLs were scattered on 18 chromosomes, individually explaining 6.6−39.5% of the phenotypic variation. Each parent provided about half of the increasing alleles: 122 from the bread wheat Forno and 127 from the spelt Oberkulmer.

QTL coincidence analysis revealed that each QTL for final grain weight was coincident with 2–13 traits of grain filling (Fig. 4; Supplementary Table S3 at *JXB* online). For carpel size, 69% of the QTLs were coincident with those for final grain weight on chromosomes 2A, 3B, 4A, 5A, 5DL, and 7B. Likewise, 75% of the QTLs for grain dry matter accumulation, 84% for grain water accumulation, and 64% for grain dimensions at maturity were coincident with those for grain weight. All coincident QTLs had the increasing alleles conferred by the same parents (Fig. 4). The exceptions were the QTLs for the onset of grain filling on chromosome 4A (the decreasing allele desired) and one QTL for carpel size on 7B (the increasing allele conferred by Oberkulmer). These QTL coincidences explained the positive physiological relationships between final grain weight and grain filling traits.

Nine QTLs for carpel size were coincident with 27 QTLs for initial, rapid, and average grain filling rates (Fig. 4; Supplementary Table S4 at *JXB* online); the increasing alleles of these QTLs (except the one for carpel size on 7B) were conferred by the same parents. Similarly, 10 and 11 QTLs for carpel size were coincident with 46 QTLs for grain water accumulation (excluding *t*_mwc_) and with 20 QTLs for final grain dimensions at maturity, respectively, with the increasing alleles conferred by the same parents (except one QTL for carpel size on 7B, and one for each of L/W and L/H on 5A).

QTL coincidences between grain water and dry matter accumulation occurred on seven chromosomes (2A, 3B, 4A, 4DL, 5A, 5DL, and 7B), including 71 of 85 QTLs for maximum grain water content and grain water absorption and loss rates, and 60 of 78 QTLs for the initial, rapid, late, average, and maximum grain filling rates (Fig. 4). Furthermore, QTL coincidences between grain water accumulation (72 of 85 QTLs) and final grain dimensions (26 of 31 QTLs for length, width, height, and volume) occurred on seven chromosomes (1BS, 2A, 3B, 4A, 5A, 5DL, and 7B). These coincident QTL had the same parents conferring the increasing alleles, confirming the positive physiological relationships among them (Table 2). Similar QTL coincidences were also observed between final grain dimensions and grain filling rates (Fig. 4).

Taken together, QTL coincidences among final grain weight, carpel size, grain dry matter and water accumulation, and final grain dimensions were found on 16 chromosomes, with the increasing alleles usually conferred by the same parents, indicating pleiotropy or the tight linkages of functionally related genes. This is consistent with their physiological relationships. Interestingly, a large number of coincident QTLs were observed on chromosomes 2A (36 QTLs for 12 traits), 3B (37 QTLs for 13 traits), 4A (39 QTLs for 14 traits), 5A (16 QTLs for 13 traits), 5DL (20 QTLs for 12 traits), and 7B (49 QTLs for 12 traits), which would offer the opportunity for improvement of multiple grain filling traits simultaneously.

### Inflection points of grain filling rate, grain dimensions, and flag leaf senescence occur at around the time of maximum grain water content (*t*_mwc_)

**Fig. 5.**
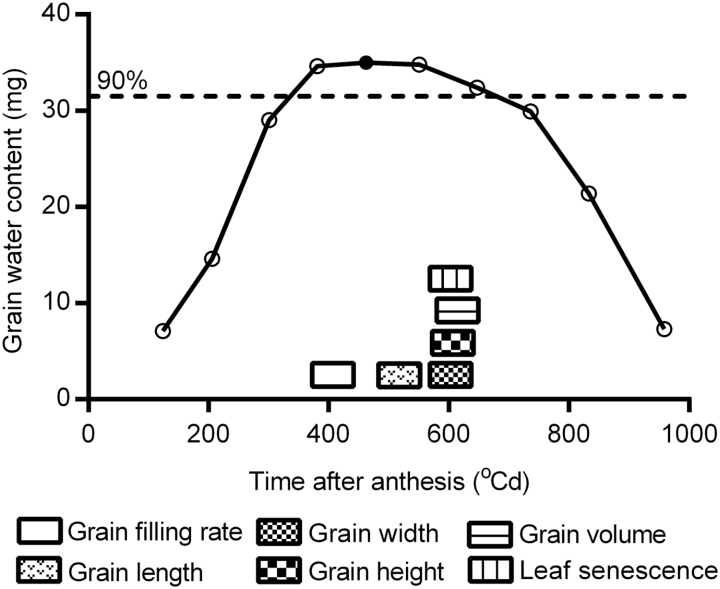
Schematic diagram of the timing of maximum grain filling rate, maximum grain water content, maximum grain dimensions and maximum senescence rate of flag leaves. Maximum grain water content is indicated by a closed circle on the curve. The horizontal dashed line indicates 90% of the maximum grain water content.

The average grain filling rate across 10 RILs and all grain positions reached its maximum first (413 DAA), followed by grain water content (500 DAA), grain length (560 DAA), grain width (603 DAA), grain height (612 DAA), and grain volume (627 DAA) (Fig. 5). Interestingly, flag leaf senescence (*t*_msr_, 591 DAA) progressed rapidly around the time for maximum grain dimensions, and there were positive relationships between *t*_msr_ and the time for maximum grain dimensions (length, *r*=0.29, *P*<0.05; width, *r*=0.48, *P*<0.01; height, *r*=0.37, *P*<0.01; and volume, *r*=0.39, *P*<0.01), indicating synchrony.

Using the grain water content as a time scale, it was found that grain filling rate reached its maximum (*t*_max_) just before *t*_mwc_ (Fig. 5). Grain expansion stopped just after grains started to lose water for desiccation, coinciding with rapid flag leaf senescence. All the events occurred around the time when 90% of the maximum grain water content was obtained (Fig. 5; Table 5).

**Table 5.** Relative grain water content at the inflection points of grain filling rate, grain dimensions, and flag leaf senescence

Grain	RGWC at *t*_max_^a^		RGWC at *t*_mgl_	RGWC at *t*_mgw_	RGWC at *t*_mgh_	RGWC at *t*_mgv_	RGWC at *t*_msr_	
2012	2013	2013	2013	2013	2013	2012	2013
Grain 1	95	96	97	94	93	92	97	88
Grain 2	95	96	98	95	93	93	97	89
Grain 3	91	95	97	94	94	91	94	91

^*a*^RGWC, relative grain water content (%), calculated as the proportion of grain water content at the inflection points to maximum water content; *t*_max_, time at the maximum grain filling rate; *t*_mgl_, time at the maximum grain length; *t*_mgw_, time at the maximum grain width; *t*_mgh_, time at the maximum grain height; *t*_mgv_, time at the maximum grain volume; *t*_msr_, time at the maximum senescence rate of flag leaves.

### Distal and basal grains within spikelets differ in grain filling processes

**Fig. 6.**
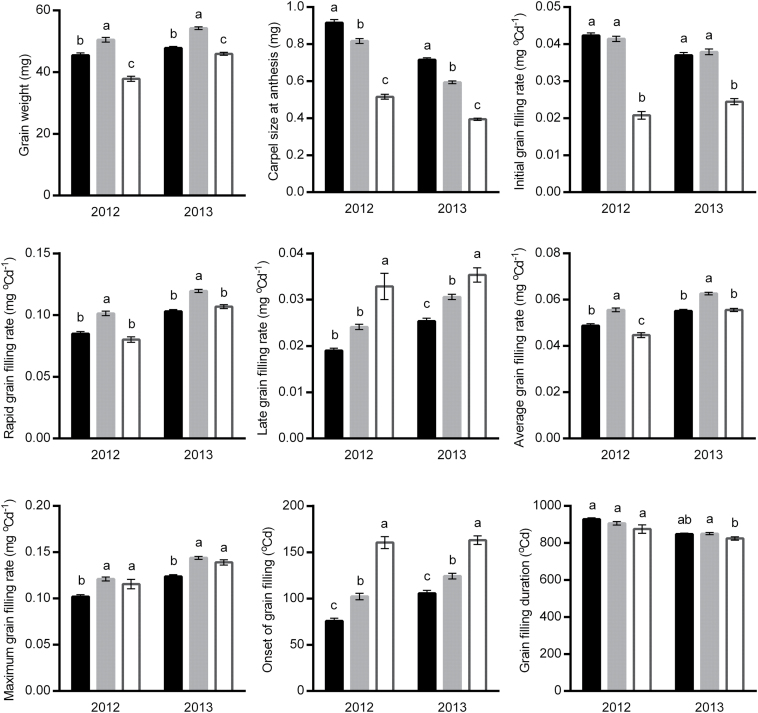

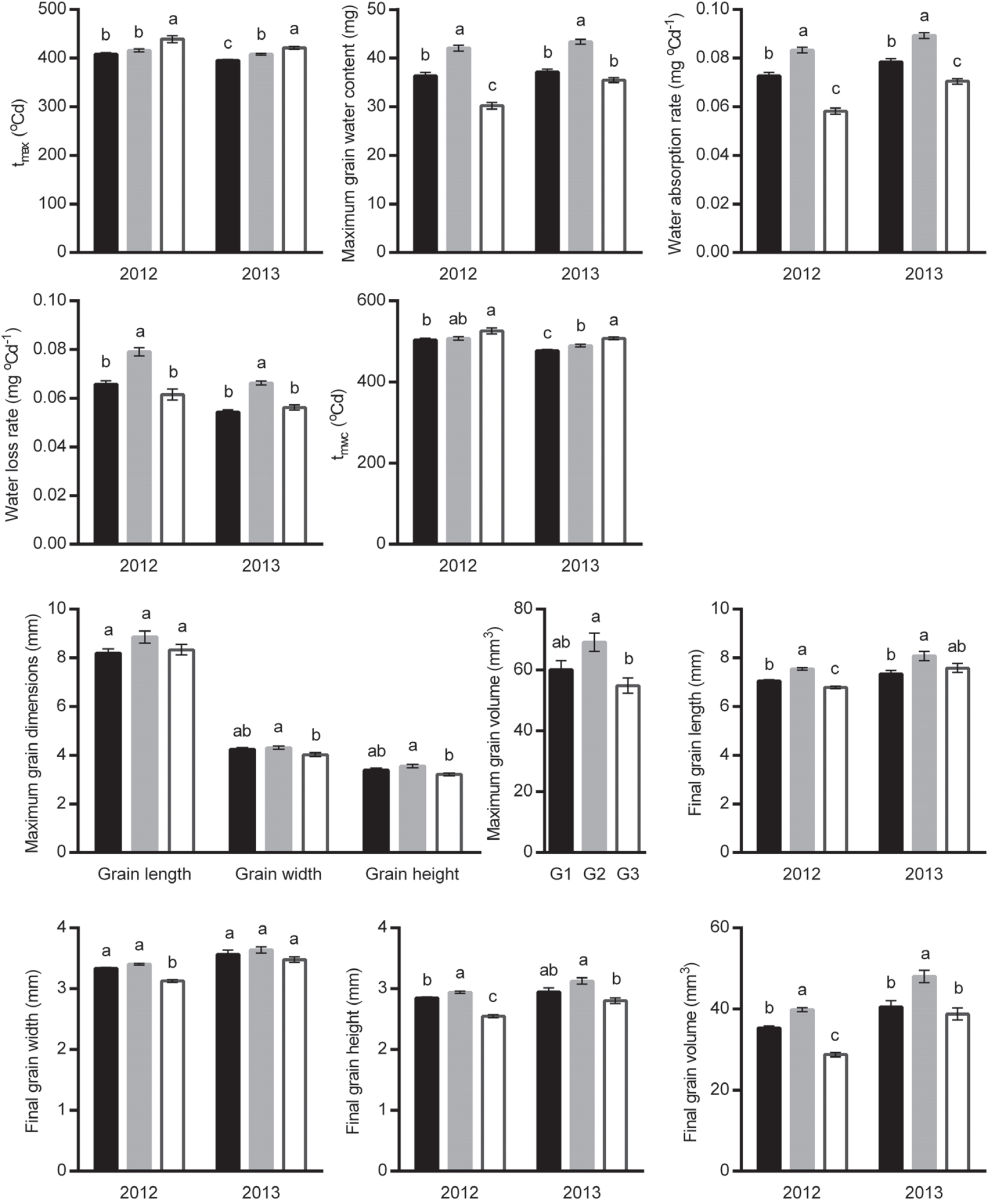
Comparisons of grain weight and grain filling traits between Grain 1, 2, and 3 within spikelets. Black, grey, and open bars (mean ±SEM) indicate Grain 1, 2, and 3, respectively. Different letters above the bars indicate significant differences between grain positions (*P*<0.01). Trait abbreviations: *t*_max_, time at the maximum grain filling rate; *t*_mwc_, time at the maximum grain water content. Comparisons of maximum grain dimensions were done in 2013 only.

As expected, G3 had lower final grain weight than G1 and G2 (Fig. 6). A further analysis showed that G3 had much smaller carpels and a slower initial grain filling rate. The rapid grain filling rate of G3 was similar to that of G1, but lower than that of G2, whereas the late grain filling rate was fastest in G3. Maximum grain filling rates of G2 and G3 were comparable, both being higher than that of G1. In addition, G3 had slower grain water absorption rate, lower maximum grain water content, and, in general, slightly smaller maximum (final) grain dimensions.

With the exception of the onset of grain filling, which was significantly later in G3, the progress of grain filling (grain filling duration, *t*_max_ and *t*_mwc_) was similar among grains (Fig. 6). Furthermore, G1, G2, and G3 reached the maximum grain dimensions at the same time (Table 6). In other words, the second half of the grain filling process (from *t*_max_) was almost synchronous among G1, G2, and G3.

**Table 6.** Timing of Grain 1, 2, and 3 at maximum grain dimensions in 2013

Grain	Timing at maximum grain dimensions (°Cd after anthesis)			
Length	Width	Height	Volume
Grain 1	554	601	605	617
Grain 2	552	593	623	618
Grain 3	573	614	608	644
*P*-value	>0.05	>0.05	>0.05	>0.05

### Distal and basal grains within spikelets differ in the genetic architectures of grain filling processes

G3 usually had fewer significant QTLs detected for grain weight, carpel size, grain dry matter, and water accumulation than G1 and G2 (Supplementary Tables S2, S5 at *JXB* online). An exception was the QTL number for the rapid grain filling rate, which was similar across the different grain positions. G1, G2, and G3 shared some QTLs for most traits, and the most common QTLs across all grain positions were located on 4A and 7B (Fig. 4; Supplementary Fig. S2). The additive effects of the shared QTLs were lower in G3 for final grain weight, maximum grain water content, and water absorption rate, but higher for carpel size, the rapid, average, and maximum grain filling rates, and grain water loss rate. There were more position-specific QTLs for G1 than for G2 or G3 (Supplementary Fig. S2).

### Distal grains respond to de-graining at anthesis

De-graining at anthesis increased the grain weight of G3 by 17% (*P*<0.05) and of G2 by 12% (*P*<0.05) across the two bread wheat cultivars Forno and Duxford. G1 did not significantly respond to de-graining, indicating that grains are more source limited from the basal to distal florets within spikelets. After de-graining, the dry weight of G3 (47.7mg) was comparable with that of G1 (48.6mg) and G2 (47.4mg) in the intact spikes (*P*>0.05).

## Discussion

### Close relationships between grain filling traits and final grain weight, and among the grain filling traits

Grain weight at maturity is a direct function of dry matter accumulation during grain filling. It was observed that the initial, rapid, average, and maximum grain filling rates, rather than the late grain filling rate, were closely associated with final grain weight. The rapid grain filling rate was three times faster than the initial and late rates, and contributed most to final grain weight. In contrast, only a weak relationship between grain filling duration and grain weight was found, indicating that the rate of synthesis of storage products is more important than its duration, consistent with previous studies (Charmet *et al.*, 2005; Wang *et al.*, 2009). Stresses such as heat, drought, and elevated CO_2_ usually stimulate the grain filling rate, but shorten its duration (Li *et al.*, 2001; Zahedi and Jenner, 2003; Yang *et al.*, 2004), suggesting the plasticity and central role of the synthetic efficiency of storage products. In addition, earlier grain filling seems to be favourable for final grain weight, as it increased the initial grain filling rate and whole grain filling duration.

A positive relationship between carpel size at anthesis and final grain weight was found, consistent with earlier reports in wheat (Calderini *et al.*, 1999; Hasan *et al.*, 2011), barley (*Hordeum vulgare* L.; Scott *et al.*, 1983), and sorghum [*Sorghum bicolor* (L.) Moench; Yang *et al.*, 2009]. A further analysis in the present study revealed that larger carpels accelerated the initial and rapid grain filling rates (mainly the former), advanced the onset of grain filling, and slightly extended grain filling duration, resulting in higher grain weight. Moreover, larger carpels increased maximum grain water content, grain water absorption and loss rates, and grain dimensions. The relationships of carpels to maximum grain water content and grain dimensions were also reported in a recent study (Hasan *et al.*, 2011). These findings indicate that final grain weight is determined during both pre- and post-anthesis periods (Calderini *et al.*, 1999). The carpel size mediates final grain weight mainly through its effects on the initial phase of grain filling.

A strong and positive relationship between maximum grain water content and final grain weight was observed in this study, as reported earlier in wheat (Lizana *et al.*, 2010; Hasan *et al.*, 2011; Gonzalez *et al.*, 2014), maize (Borras *et al.*, 2003; Sala *et al.*, 2007), and sunflower (Rondanini *et al.*, 2009). Further, grain water absorption and loss rates were also positively associated with final grain weight. These contributions probably resulted from the effects of grain water accumulation on the accelerated grain filling rates. Similarly, grain water accumulation and maximum (final) grain dimensions were positively associated. To understand the potential roles of grain water uptake and loss during grain filling, the timing of key grain growth events was compared in detail. The results showed that the grain filling rate reached its maximum while the grain water content levelled off, which was also observed in the reports of Laudencia-Chingcuanco *et al.* (2007) and Lizana *et al.* (2010). Considering the positive relationships between maximum grain water content and maximum grain filling rate, and between grain water absorption rate and the rates of initial and rapid grain filling, it can be deduced that grain water drives the synthesis of storage products, serving as a raw material or medium. In addition, grain length reached its maximum just after maximum grain water content. Following this, grain width, height, and volume stopped expanding almost simultaneously, while the grains started to lose water. This implies that grain water may function as an incentive for grain dimension establishment. Once grain desiccation commences, the driving force disappears and grain enlargement ends. Briarty *et al.* (1979) reported that the endosperm and cell volume reach their maximums at the same time (35 d after anthesis), and the timing is similar to that for maximum grain water content in this study (31 d), supporting the above hypothesis. Meanwhile, the flag leaves also underwent rapid senescence, indicating synchrony. Around this critical time, rapid reduction in flag leaf and ear photosynthesis (Sofield *et al.*, 1977) and programmed cell death in the entire endosperm of grains (Young and Gallie, 1999) occur. Taken together, it seems that there is a critical time when multiple organs (grain, ear, and flag leaf) undergo rapid senescence simultaneously. Expression of the genes for dehydrins, late embryo abundant proteins, and tritins peaks at this time (Laudencia-Chingcuanco *et al.*, 2007), implying their possible roles in regulating the synchronous senescence processes.

### QTL coincidences reflect the close relationships between carpel size, grain dry matter and water accumulation, grain morphology, and final grain weight

Grain filling is a complex but orderly process. QTL analysis revealed a large number of QTLs for the grain filling traits. Of them, the QTLs for carpel size, grain filling rates for different phases, and grain water uptake and loss are reported here for the first time in wheat. Each QTL for grain weight was coincident with 2–13 traits of grain filling; 73%, on average, of the QTLs for the grain filling traits were coincident with those for grain weight, with the favourable alleles usually conferred by the same parents. The high level of QTL coincidences was also found among the grain filling traits. These QTL coincidences confirm the roles of carpel size, grain dry matter accumulation, grain water uptake and loss, and grain dimensions as the physiological determinants of final grain weight, and also explain the close relationships between the grain filling traits. The orderly processes of grain filling therefore result mainly from the pleiotropy or tight linkages of functionally related genes. Across the whole genome, a limited number of QTL clusters were identified on six chromosomes (2A, 3B, 4A, 5A, 5DL, and 7B), and each of them harboured 16–49 QTLs for >12 traits. They offer an opportunity to improve multiple grain filling traits simultaneously in wheat breeding.

QTL coincidences between grain filling rate and duration, and final grain weight have also been identified in an earlier study (Wang *et al.*, 2009), in which the QTL clusters reported on 3B correspond approximately to those in the present study. In addition, the QTL coincidences between final grain dimensions and grain weight have been observed on many chromosomes (1B, 2A, 2B, 2D, 3A, 3B, 4B, 4D, 5A, 6A, and 7A) (Breseghello and Sorrells, 2007; Gegas *et al.*, 2010; Williams *et al.*, 2013). In this study, 16 QTLs for 13 traits and 20 QTLs for 12 traits (most of them for G1) were detected on homoeologous chromosomes 5AL and 5DL, respectively, suggesting homoeoalleles with similar functions. Meanwhile, the QTLs for grain threshability and glume tenacity (domestication traits) were identified on 5AL as well (data not shown). Common marker analysis showed that these QTLs correspond to the domestication gene *Q* (Kato *et al.*, 1998; Simonetti *et al.*, 1999), with the bread wheat Forno providing the free-threshing allele *Q*. This implies that the *Q* allele in bread wheat may be associated with higher grain weight of G1 and other favourable traits of grain filling.

### Late onset of grain filling and slow initial grain filling rate lead to smaller distal grains

Onset of grain filling was much later in G3 than in G1 and G2, and the subsequent progress of grain filling was almost synchronous among grains. Simultaneous termination of dry matter and water accumulation, as well as the coincidence of rapid grain desiccation among G1, G2, and G3 have also been found in other studies (Simmons and Crookston, 1979; Millet and Pinthus, 1984). The synchrony at late grain filling is probably as a result of the whole plant senescence for maturation (as discussed above).

Compared with G1 and G2, the initial grain filling rate was much slower in G3. This may result from the late onset of grain filling, as there was a strong negative relationship between them. In contrast, G3 had a rapid grain filling rate similar to G1, and also showed a fast maximum grain filling rate, indicating that G3 is capable of rapid dry matter accumulation like G1 and G2. This can be supported by the genetic evidence, which showed that G3 had a similar number of QTLs for the rapid grain filling rate, and that the additive effects of the shared QTLs for the rapid and maximum grain filling rates were even higher in G3. It has been observed that the maximum starch synthetic rate and some enzyme activities are comparable or even higher in distal grains than in basal grains (Jiang *et al.*, 2003). In addition, G3 had a significantly higher late grain filling rate. Despite the capability for efficient dry matter accumulation after the initial phase of grain filling, G3 could not be fully filled because of the synchronous senescence of grains and other organs.

The combination of late onset of grain filling and a slow initial grain filling rate could be responsible for the smaller G3. This may be explained by a delay of 2–5 d for anthesis of distal florets compared with basal ones (Simmons and Crookston, 1979; Millet and Pinthus, 1984), and the resultant later carpel growth and smaller carpel size at anthesis (Calderini *et al.*, 1999). Moreover, a greater increase in dry weight of distal grains than of basal grains was found after de-graining in the present and previous studies (Simmons *et al.*, 1982; Calderini and Reynolds, 2000; Acreche and Slafer, 2009), indicating that distal grains are more source limited, whereas basal grains are more sink limited. In many cases, final grain weight can be comparable or even higher in distal grains than in basal grains after de-graining (Radley, 1978; Simmons *et al.*, 1982; Gao *et al.*, 1992; Calderini and Reynolds, 2000). These results thus indicate that distal grains can be improved through increased assimilate supply. As the grain filling rate is sufficient in G3 during the rapid and late phases, the increased assimilate availability after de-graining may have improved the initiation of grain filling (rate and onset). Evidence can be found from the de-graining treatment at heading, which significantly accelerates floret development of G3 and increases carpel size at anthesis (Calderini and Reynolds, 2000). De-graining immediately after anthesis can also improve the onset and rate of initial dry matter and water accumulation, and finally produce a higher dry weight of G3 (Radley, 1978; Gao *et al.*, 1992). Limited assimilate availability for G3 during the initial grain filling phase, under normal growing conditions, probably results from the priority for assimilate partitioning to basal grains.

### Conclusions

Individual grain weight is an important but complex trait in wheat. This study showed that the pre-anthesis carpel growth and post-anthesis grain dry matter and water accumulation, as well as grain morphological expansion, are closely associated with each other, and with final grain weight. Genetic analysis demonstrated a high level of QTL coincidences between these traits, indicating pleiotropy or the tight linkages of functionally related genes. Frequent QTL coincidences, particularly those on chromosomes 2A, 3B, 4A, 5A, 5DL, and 7B, will be useful to improve multiple grain filling traits simultaneously through marker-assisted breeding. In future work, the candidate genes residing in these narrow QTL regions will be identified by fine mapping based on the next-generation sequencing technologies. This process can be complemented by syntenic studies with other species such as rice and barley. In addition, there is a great variation in grain weight within spikelets, and smaller distal grains stem mainly from later grain filling and a slower initial grain filling rate, compared with the basal grains. Although distal grains are capable of rapid dry matter accumulation thereafter, they cannot be fully filled because of the synchronous maturation or terminal plant senescence. An increase in assimilate availability around anthesis is able to improve the distal grain weight. Therefore, this study helps to understand grain weight determination in wheat. Spelt is an old-world, hexaploid relative of bread wheat. As presented here, the spelt Oberkulmer showed a number of desirable physiological traits to improve grain weight, including larger carpels, longer grains, and larger grain volume. Many favourable alleles associated with the grain filling traits were also determined. These demonstrate that spelt may be used to broaden the genetic diversity of bread wheat in terms of grain weight improvement.

## Supplementary data

Supplementary data are available at *JXB* online.

Figure S1. Grain expansion dynamics in 2013.

Figure S2. Quantitative trait locus (QTL) comparisons between different grains within spikelets.

Table S1. Descriptive statistics on the final grain weight and grain filling traits of the parents and recombinant inbred line (RIL) mapping population.

Table S2. Quantitative trait locus (QTL) identification for grain weight, carpel size, grain dry matter and water accumulation, and grain dimensions.

Table S3. Quantitative trait locus (QTL) coincidences between final grain weight and grain filling traits.

Table S4. Quantitative trait locus (QTL) coincidences between carpel size and the other grain filling traits.

Table S5. Quantitative trait locus (QTL) number detected for Grain 1, 2, and 3.

Supplementary Data

## Abbreviations:

C1 (C2 or C3): the first (second or third) carpel within a spikelet counting from the rachis
DAA: degree days after anthesis
G1 (G2 or G3): the first (second or third) grain within a spikelet counting from the rachis
*H*^2^: broad sense heritability
LOD: logarithm of the odds
L/H: grain length/height
L/W: grain length/width
QTL: quantitative trait locus
RIL: recombinant inbred line
*t*_max_: the time at maximum grain filling rate
*t*_mwc_: the time at maximum grain water content.
