# Supplementary material for: Carpel size, grain filling, and morphology determine individual grain weight in wheat
Source: J Exp Bot. 2015 Aug 5;66(21):6715–30. doi: 10.1093/jxb/erv378 (PMC4623684; doi:10.1093/jxb/erv378)
Supplement: Supplementary Data [file supp_66_21_6715__index.html]

Carpel size, grain filling, and morphology determine individual grain weight in wheat — Carpel size, grain filling, and morphology determine individual grain weight in wheat — Supplementary Data 

# Carpel size, grain filling, and morphology determine individual grain weight in wheat

## Supplementary Data

Data files

- Supplementary Data - Supplementary Data
