# Supplementary material for: Carpel size, grain filling, and morphology determine individual grain weight in wheat
Source: J Exp Bot. 2015 Aug 5;66(21):6715–30. doi: 10.1093/jxb/erv378 (PMC4623684; doi:10.1093/jxb/erv378)
Supplement: Supplementary Data [file supp_erv378_jexbot151548_file001.pdf]

Supplementary data

Carpel size, grain filling and morphology determine individual grain weight in wheat

Quan Xie, Sean Mayes and Debbie L. Sparkes

Supplementary Table S1. Descriptive statistics on the final grain weight and grain filling traits of the parents and recombinant inbred line (RIL) mapping population.

Supplementary Table S2. Quantitative trait locus (QTL) identification for grain weight, carpel size, grain dry matter and water accumulation, and grain dimensions.

Supplementary Table S3. Quantitative trait locus (QTL) coincidences between final grain weight and grain filling traits.

Supplementary Table S4. Quantitative trait locus (QTL) coincidences between carpel size and the other grain filling traits.

Supplementary Table S5. Quantitative trait locus (QTL) number detected for Grain 1, 2 and 3.

Supplementary Fig. S1. Grain expansion dynamics in 2013.

Supplementary Fig. S2. Quantitative trait locus (QTL) comparisons between different grains within spikelets.

Supplementary Table S1. Descriptive statistics on the final grain weight and grain filling traits of the parents and recombinant inbred line (RIL) mapping population

Trait[a]	Year	Parental lines			RILs			H^{2c}
		Forno	Oberkulmer	P-value	Mean (min; max)	P-value	SED[b]	
**Final grain weight**								
Grain 1 (mg)	2012	46.0	49.7	> 0.05	45.4 (30.5; 63.0)	< 0.001	3.4	0.81
	2013	57.3	45.7	< 0.01	47.8 (35.5; 60.4)	< 0.001	1.7	
Grain 2 (mg)	2012	44.1	58.8	< 0.01	50.5 (38.0; 65.1)	< 0.001	4.1	0.70
	2013	57.3	55.9	> 0.05	54.2 (45.0; 66.1)	< 0.001	2.0	
Grain 3 (mg)	2012	32.7	39.7	> 0.05	37.9 (17.8; 51.5)	< 0.001	5.7	0.67
	2013	49.2	44.8	> 0.05	45.9 (33.6; 58.3)	< 0.001	2.6	
**Carpel size at anthesis**								
Carpel 1 (mg)	2012	0.73	1.07	< 0.01	0.91 (0.57; 1.27)	< 0.001	0.12	0.61
	2013	0.60	0.87	< 0.01	0.72 (0.48; 1.09)	< 0.001	0.08	
Carpel 2 (mg)	2012	0.66	0.95	< 0.01	0.82 (0.57; 1.12)	< 0.001	0.10	0.41
	2013	0.46	0.67	< 0.01	0.59 (0.45; 0.80)	< 0.001	0.06	
Carpel 3 (mg)	2012	0.36	0.53	< 0.05	0.52 (0.23; 0.78)	< 0.001	0.08	0.38
	2013	0.40	0.45	> 0.05	0.39 (0.27; 0.59)	< 0.001	0.05	
**Grain dry matter accumulation**								
**Grain 1**								
Initial GFR (mg $^oCd^{-1}$)	2012	0.047	0.047	> 0.05	0.042 (0.026; 0.056)	< 0.001	0.005	0.28
	2013	0.043	0.025	< 0.05	0.037 (0.011; 0.055)	< 0.001	0.008	
Rapid GFR (mg $^oCd^{-1}$)	2012	0.085	0.090	> 0.05	0.085 (0.052; 0.135)	< 0.001	0.011	0.72
	2013	0.123	0.096	< 0.05	0.103 (0.064; 0.151)	< 0.001	0.011	
Late GFR (mg $^oCd^{-1}$)	2012	0.018	0.020	> 0.05	0.019 (0.011; 0.035)	< 0.001	0.003	0.43
	2013	0.030	0.027	> 0.05	0.025 (0.013; 0.074)	< 0.001	0.006	
Average GFR (mg $^oCd^{-1}$)	2012	0.050	0.052	> 0.05	0.049 (0.031; 0.070)	< 0.001	0.004	0.84
	2013	0.065	0.049	< 0.01	0.055 (0.040; 0.079)	< 0.001	0.003	
Maximum GFR (mg $^oCd^{-1}$)	2012	0.103	0.108	> 0.05	0.102 (0.063; 0.161)	< 0.001	0.012	0.70
	2013	0.146	0.117	< 0.05	0.124 (0.081; 0.229)	< 0.001	0.014	
Onset of GF (^oCd)	2012	61	76	> 0.05	76 (25; 154)	< 0.05	29	0.25
	2013	114	151	> 0.05	105 (0; 236)	< 0.001	39	
GF duration (^oCd)	2012	906	943	> 0.05	928 (783; 1094)	< 0.05	70	0.21
	2013	857	883	> 0.05	847 (690; 1082)	< 0.001	63	
t_{max} (^oCd)	2012	391	415	> 0.05	408 (356; 481)	< 0.01	24.7	0.52
	2013	404	437	< 0.05	395 (355; 469)	< 0.001	15	

[a] Trait abbreviations: GFR, grain filling rate; GF, grain filling; t_{max}, the time at maximum grain filling rate; t_{mwc}, the time at maximum grain water content.

[b] SED: standard error of the difference of mean.

[c] H^2: broad sense heritability.

Supplementary Table S1. (continued)

Trait	Year	Parental lines: Forno	Oberkulmer	P-value	RILs: Mean (min; max)	P-value	SED	H^2
**Grain 2**								
Initial GFR (mg $^oCd^{-1}$)	2012	0.042	0.050	> 0.05	0.041 (0.026; 0.056)	< 0.05	0.007	0.20
	2013	0.046	0.025	< 0.05	0.038 (0.020; 0.056)	< 0.001	0.008	
Rapid GFR (mg $^oCd^{-1}$)	2012	0.093	0.109	> 0.05	0.101 (0.063; 0.130)	< 0.001	0.014	0.71
	2013	0.124	0.123	> 0.05	0.120 (0.090; 0.147)	< 0.001	0.012	
Late GFR (mg $^oCd^{-1}$)	2012	0.022	0.025	> 0.05	0.024 (0.014; 0.035)	< 0.01	0.005	0.49
	2013	0.029	0.035	> 0.05	0.031 (0.020; 0.054)	< 0.05	0.006	
Average GFR (mg $^oCd^{-1}$)	2012	0.052	0.061	< 0.05	0.056 (0.038; 0.069)	< 0.001	0.004	0.80
	2013	0.066	0.061	> 0.05	0.063 (0.049; 0.075)	< 0.001	0.004	
Maximum GFR (mg $^oCd^{-1}$)	2012	0.111	0.130	> 0.05	0.121 (0.076; 0.155)	< 0.001	0.016	0.69
	2013	0.146	0.149	> 0.05	0.144 (0.107; 0.195)	< 0.001	0.017	
Onset of GF (^oCd)	2012	80	98	> 0.05	103 (32; 179)	< 0.001	29	0.35
	2013	104	187	< 0.05	124 (37; 202)	< 0.05	36	
GF duration (^oCd)	2012	839	953	> 0.05	906 (711; 1104)	< 0.01	79	0.24
	2013	853	874	> 0.05	850 (694; 1022)	< 0.001	62	
t_{max} (^oCd)	2012	376	432	< 0.05	416 (346; 475)	< 0.001	26	0.46
	2013	396	455	< 0.01	408 (363; 490)	< 0.001	18	
**Grain 3**								
Initial GFR (mg $^oCd^{-1}$)	2012	0.026	0.002	< 0.01	0.021 (0.000; 0.036)	< 0.001	0.008	0.27
	2013	0.033	0.013	< 0.05	0.025 (0.000; 0.049)	< 0.001	0.009	
Rapid GFR (mg $^oCd^{-1}$)	2012	0.070	0.070	> 0.05	0.080 (0.036; 0.118)	< 0.001	0.015	0.45
	2013	0.119	0.103	> 0.05	0.107 (0.057; 0.141)	< 0.001	0.013	
Late GFR (mg $^oCd^{-1}$)	2012	0.019	0.155	< 0.01	0.031 (0.007; 0.100)	< 0.001	0.018	0.31
	2013	0.030	0.037	> 0.05	0.035 (0.015; 0.132)	< 0.001	0.011	
Average GFR (mg $^oCd^{-1}$)	2012	0.039	0.076	< 0.01	0.044 (0.024; 0.063)	< 0.001	0.007	0.59
	2013	0.061	0.051	> 0.05	0.056 (0.039; 0.073)	< 0.001	0.005	
Maximum GFR (mg $^oCd^{-1}$)	2012	0.088	0.323	< 0.01	0.113 (0.046; 0.217)	< 0.001	0.042	0.42
	2013	0.141	0.135	> 0.05	0.139 (0.085; 0.272)	< 0.001	0.029	
Onset of GF (^oCd)	2012	92	268	< 0.01	160 (21; 291)	< 0.001	50	0.39
	2013	138	237	< 0.05	162 (0; 310)	< 0.001	46	
GF duration (^oCd)	2012	862	526	< 0.05	880 (510; 1555)	< 0.001	135	0.25
	2013	813	846	> 0.05	824 (576; 1139)	< 0.001	94	
t_{max} (^oCd)	2012	392	369	> 0.05	441 (331; 676)	< 0.01	55	0.26
	2013	401	475	< 0.01	421 (328; 501)	< 0.001	26	
**Grain water accumulation**								
**Grain 1**								
Maximum grain water content (mg)	2012	36.4	37.7	> 0.05	36.3 (22.9; 52.2)	< 0.001	2.1	0.91
	2013	48.7	31.8	< 0.01	37.1 (25.3; 56.6)	< 0.001	1.8	
Water absorption rate (mg $^oCd^{-1}$)	2012	0.079	0.067	< 0.05	0.073 (0.049; 0.104)	< 0.001	0.005	0.87
	2013	0.099	0.061	< 0.01	0.078 (0.046; 0.119)	< 0.001	0.005	
Water loss rate (mg $^oCd^{-1}$)	2012	0.055	0.081	< 0.01	0.066 (0.034; 0.092)	< 0.001	0.010	0.75
	2013	0.069	0.052	< 0.01	0.054 (0.037; 0.084)	< 0.001	0.004	
t_{mwc} (^oCd)	2012	458	565	< 0.01	503 (426; 584)	< 0.001	36	0.17
	2013	492	523	> 0.05	476 (415; 603)	< 0.001	20	

Supplementary Table S1. (continued)

Trait	Year	Parental lines			RILs			H^2
		Forno	Oberkulmer	P-value	Mean (min; max)	P-value	SED	
**Grain 2**								
Maximum grain water content (mg)	2012	38.1	47.5	< 0.01	42.0 (29.0; 54.0)	< 0.001	2.7	0.86
	2013	51.8	41.0	< 0.01	43.3 (32.1; 55.0)	< 0.001	2.3	
Water absorption rate (mg $^{o}Cd^{-1}$)	2012	0.089	0.082	> 0.05	0.083 (0.060; 0.109)	< 0.001	0.008	0.81
	2013	0.107	0.071	< 0.01	0.089 (0.059; 0.125)	< 0.001	0.006	
Water loss rate (mg $^{o}Cd^{-1}$)	2012	0.057	0.110	< 0.01	0.079 (0.047; 0.114)	< 0.001	0.013	0.70
	2013	0.073	0.076	> 0.05	0.066 (0.049; 0.086)	< 0.001	0.005	
t_{mwc} (^{o}Cd)	2012	429	582	< 0.01	508 (431; 583)	< 0.001	39	0.52
	2013	483	575	< 0.01	489 (425; 596)	< 0.001	25	
**Grain 3**								
Maximum grain water content (mg)	2012	26.4	43.8	< 0.01	30.1 (13.5; 41.7)	< 0.001	4.6	0.67
	2013	43.6	30.2	< 0.01	35.5 (23.5; 50.7)	< 0.001	2.8	
Water absorption rate (mg $^{o}Cd^{-1}$)	2012	0.060	0.068	> 0.05	0.058 (0.033; 0.080)	< 0.001	0.011	0.43
	2013	0.089	0.050	< 0.01	0.070 (0.043; 0.101)	< 0.001	0.008	
Water loss rate (mg $^{o}Cd^{-1}$)	2012	0.042	0.134	< 0.01	0.061 (0.019; 0.108)	< 0.001	0.015	0.65
	2013	0.062	0.062	> 0.05	0.056 (0.036; 0.115)	< 0.001	0.010	
t_{mwc} (^{o}Cd)	2012	442	647	< 0.01	525 (391; 688)	< 0.001	61	0.43
	2013	494	616	< 0.01	507 (427; 652)	< 0.001	42	
**Grain dimensions at maturity**								
Grain length (mm)	2012	6.5	7.8	< 0.01	7.2 (6.2; 8.2)	< 0.001	0.2	0.79
	2013	7.0	8.2	< 0.01	7.5 (6.3; 8.6)	< 0.001	0.3	
Grain width (mm)	2012	3.4	3.2	> 0.05	3.3 (3.0; 3.6)	< 0.001	0.1	0.55
	2013	3.3	3.1	> 0.05	3.2 (2.9; 3.5)	< 0.001	0.1	
Grain height (mm)	2012	2.8	2.8	> 0.05	2.8 (2.5; 3.2)	< 0.001	0.09	0.73
	2013	3.0	2.8	> 0.05	2.8 (2.5; 3.3)	< 0.001	0.1	
Grain volume (mm^3)	2012	32.4	37.8	< 0.05	35.3 (27.0; 43.9)	< 0.001	2.4	0.68
	2013	35.6	37.8	> 0.05	35.3 (26.8; 47.8)	< 0.001	2.7	
Grain length/width	2012	1.9	2.4	< 0.01	2.2 (1.9; 2.4)	< 0.001	0.07	0.81
	2013	2.1	2.6	< 0.01	2.4 (2.0; 2.9)	< 0.001	0.1	
Grain length/height	2012	2.3	2.8	< 0.01	2.6 (2.2; 2.9)	< 0.001	0.08	0.79
	2013	2.3	2.9	< 0.01	2.6 (2.2; 3.2)	< 0.001	0.1	

Supplementary Table S2. Quantitative trait locus (QTL) identification for grain weight, carpel size, grain dry matter and water accumulation, and grain dimensions

Trait/Chromosome	Year	Position (cM)	LOD	R^{2a}	Additive effect[b]	Closest marker
**Final grain weight**						
**Grain 1**						
2A	2012	94.9	3.14	18.7	-3.02	*Xpsr919b-2A*
	2013	142.1	3.14	12.3	-1.86	*Xglk699b-2AL*
2B	2013	145.8	3.16	12.4	-1.83	*Xglk699a-2BS*
3B	2013	1.0	4.76	18.1	2.36	*Xpsr1327a-3B*
4A	2012	21.7	5.54	30.5	-4.52	*Xpsr59a-4A*
5A	2013	209.9	8.70	30.5	3.37	*Xpsr918b-5A*
5DL	2012	36.0	3.55	20.8	-6.71	*Xpsr580a-5DL*
	2013	48.0	5.48	20.5	-4.45	*Xpsr580a-5DL*
7B	2012	70.7	3.54	20.8	3.29	*Xglk549-7B*
	2012	189.5	3.75	21.9	4.82	*Xmwg710a-7B*
7D	2013	84.8	4.06	15.6	2.45	*Xpsr662-7D*
**Grain 2**						
2A	2013	143.4	3.20	12.6	-1.62	*PL_AP-2A*
3B	2012	80.5	4.17	24.0	3.49	*Xpsr1054-3B*
	2013	1.9	3.66	14.2	1.76	*C970a-3B*
	2013	80.5	5.47	20.5	2.14	*Xpsr1054-3B*
4A	2012	21.7	5.80	31.7	-4.53	*Xpsr59a-4A*
	2013	32.2	4.01	15.4	-2.10	*Xpsr59a-4A*
7B	2012	217.4	3.56	20.9	3.10	*Xglk576-7BL*
7D	2013	92.8	3.37	13.2	1.84	*Xgwm111b-7D*
**Grain 3**						
2A	2013	90.9	4.91	18.6	-2.28	*Xpsr151-2A*
3B	2013	80.5	3.38	13.2	2.03	*Xpsr1054-3B*
4A	2012	21.7	4.16	23.9	-4.14	*Xpsr59a-4A*
	2013	24.7	4.72	17.9	-2.64	*Xpsr59a-4A*
5A	2013	227.4	3.29	12.9	-2.18	*Xpsr1201a-5A*
7B	2012	217.0	3.46	20.3	3.22	*Xglk576-7BL*
	2013	15.6	3.28	12.8	2.16	*Xpsr952-7B*
**Carpel size**						
**Carpel 1**						
2A	2013	153.5	2.95	11.6	-0.04	*Xglk278a-2AL*
3B	2013	1.9	5.36	20.1	0.05	*C970a-3B*
4A	2012	27.2	4.07	23.5	-0.079	*Xpsr59a-4A*
5A	2013	210.9	6.20	22.9	0.06	*Xpsr918b-5A*
5B	2013	7.9	3.32	13.0	-0.05	*Xwg669-5B*
5DL	2013	67.8	4.62	17.6	-0.05	*Xpsr580a-5DL*
6A	2013	4.0	3.75	14.5	-0.05	*Xpsr563a-6A*

[a] R^2: the proportion of phenotypic variation explained by individual QTL.

[b] Positive additive effects indicate that the alleles from Forno increase the values of the traits, whereas negative additive effects indicate that the alleles from Oberkulmer increase the values of the traits.

Supplementary Table S2. (continued)

Trait/Chromosome	Year	Position (cM)	LOD	R^2	Additive effect	Closest marker
**Carpel 2**						
3B	2013	1.9	4.30	16.5	0.03	*C970a-3B*
4A	2012	27.2	3.78	22.0	-0.060	*Xpsr59a-4A*
5A	2012	58.3	3.29	19.4	0.066	*Xglk424-5A*
5DL	2013	67.7	3.60	14.0	-0.03	*Xpsr580a-5DL*
**Carpel 3**						
5A	2012	62.3	4.32	24.8	0.073	*Xglk424-5A*
7B	2012	51.9	3.93	22.8	-0.061	*Xpsr690-7B*
**Grain dry matter accumulation**						
**Grain 1**						
Initial grain filling rate						
2A	2012	125.7	2.90	17.0	-0.0038	*Xglk699b-2AL*
4A	2012	33.2	3.37	19.9	-0.0032	*Xpsr914-4A*
5A	2012	212.9	2.90	17.3	0.0030	*Xpsr918b-5A*
5DL	2012	40.0	2.93	17.5	-0.0054	*Xpsr580a-5DL*
7B	2012	194.5	3.22	19.1	0.0039	*Xmwg710a-7B*
Rapid grain filling rate						
1A	2012	80.1	3.28	19.4	-0.0071	*Xpsr1327b-1A*
1DS	2013	0.1	3.38	13.2	0.0057	*Xpsr168-1DS*
2A	2013	142.1	3.19	12.5	-0.0055	*Xglk699b-2AL*
3B	2013	13.1	3.35	13.1	0.0065	*Xpsr1196b-3B*
	2013	51.9	3.40	13.3	0.0059	*Xpsr1101b-3B*
4A	2012	18.7	3.04	18.1	-0.0077	*Xglk315-4AS*
5A	2013	211.9	7.64	27.4	0.0092	*Xpsr918b-5A*
5DL	2013	40.0	5.54	20.7	-0.0147	*Xpsr580a-5DL*
Late grain filling rate						
1A	2012	80.1	3.33	19.7	-0.0020	*Xpsr1327b-1A*
5A	2013	213.4	4.25	16.3	0.0030	*Xpsr918b-5A*
5DL	2013	32.0	2.73	10.8	-0.0048	*Xpsr906a-5DL*
Average grain filling rate						
1A	2012	80.1	3.04	18.1	-0.0033	*Xpsr1327b-1A*
1DS	2013	0.1	3.30	12.9	0.0027	*Xpsr168-1DS*
2A	2012	94.9	2.90	17.1	-0.0032	*Xpsr919b-2A*
	2013	142.1	3.23	12.7	-0.0026	*Xglk699b-2AL*
3B	2013	11.1	4.01	15.5	0.0034	*Xpsr1196b-3B*
	2013	51.9	3.11	12.2	0.0026	*Xpsr1101b-3B*
4A	2012	19.7	3.86	22.4	-0.0042	*Xglk315-4AS*
5A	2013	210.9	9.41	32.6	0.0048	*Xpsr918b-5A*
5DL	2012	39.0	3.34	19.7	-0.0071	*Xpsr580a-5DL*
	2013	39.0	5.71	21.2	-0.0071	*Xpsr580a-5DL*
7B	2012	70.7	3.05	18.2	0.0034	*Xglk549-7B*
	2012	187.5	3.07	18.3	0.0049	*Xglk750-7BL*

Supplementary Table S2. (continued)

Trait/Chromosome	Year	Position (cM)	LOD	R^2	Additive effect	Closest marker
Maximum grain filling rate						
1A	2012	80.1	3.27	19.4	-0.0082	*Xpsr1327b-1A*
2A	2013	142.1	3.10	12.2	-0.0072	*Xglk699b-2AL*
3B	2013	13.1	3.10	12.2	0.0083	*Xpsr1196b-3B*
	2013	51.9	3.06	12.0	0.0073	*Xpsr1101b-3B*
4A	2012	18.7	3.35	19.8	-0.0093	*Xglk315-4AS*
5A	2013	213.4	7.56	27.1	0.0116	*Xpsr918b-5A*
5DL	2013	37.0	4.74	18.0	-0.0184	*Xpsr580a-5DL*
**Grain 2**						
Rapid grain filling rate						
2A	2012	94.9	3.90	22.6	-0.0079	*Xpsr919b-2A*
3B	2013	56.9	4.88	18.5	0.0066	*Xpsr1101b-3B*
4A	2012	18.7	3.62	21.2	-0.0087	*Xglk315-4AS*
7B	2012	190.5	3.51	20.6	0.0112	*Xmwg710a-7B*
	2013	74.6	3.22	12.6	0.0052	*Xglk478-7BL*
	2013	184.5	3.47	13.5	0.0075	*Xglk750-7BL*
Late grain filling rate						
3B	2013	57.9	3.26	12.8	0.0024	*Xpsr116a-3B*
Average grain filling rate						
2A	2012	94.9	4.01	23.2	-0.0035	*Xpsr919b-2A*
	2013	90.6	3.75	14.5	-0.0023	*Xpsr151-2A*
	2013	130.6	3.38	13.2	-0.0030	*Xglk699b-2AL*
2D	2013	41.9	3.04	11.9	0.0048	*Xpsr933b-2D*
3B	2012	80.5	3.43	20.2	0.0034	*Xpsr1054-3B*
	2013	12.1	3.63	14.1	0.0027	*Xpsr1196b-3B*
	2013	77.3	4.27	16.4	0.0027	*Xpsr1054-3B*
4A	2012	19.7	4.70	26.6	-0.0044	*Xglk315-4AS*
7B	2012	190.5	4.05	23.4	0.0052	*Xmwg710a-7B*
	2013	76.6	3.97	15.3	0.0027	*Xglk478-7BL*
	2013	183.5	4.12	15.8	0.0035	*Xglk750-7BL*
Maximum grain filling rate						
2A	2012	94.9	3.69	21.5	-0.0089	*Xpsr919b-2A*
3B	2013	56.9	4.80	18.2	0.0083	*Xpsr1101b-3B*
4A	2012	18.7	3.81	22.2	-0.0103	*Xglk315-4AS*
7B	2012	191.5	3.34	19.7	0.0125	*Xmwg710a-7B*
	2013	74.6	3.20	12.5	0.0066	*Xglk478-7BL*
	2013	185.5	2.90	11.4	0.0088	*Xglk750-7BL*
Onset of grain filling						
4A	2012	0.1	3.54	20.8	-15	*Xglk752-4AS*
Grain filling duration						
3A	2013	107.3	3.52	13.7	25.9	*Xglk577-3AL*

Supplementary Table S2. (continued)

Trait/Chromosome	Year	Position (cM)	LOD	R^2	Additive effect	Closest marker
**Grain 3**						
Rapid grain filling rate						
1A	2012	62.8	3.26	19.3	-0.0147	*Xpsr1327b-1A*
2A	2012	97.9	3.05	18.2	-0.0085	*Xglk293b-2AL*
	2013	88.9	3.44	13.4	-0.0062	*Xpsr386c-2A*
	2013	127.6	4.24	16.2	-0.0099	*Xglk699b-2AL*
3B	2013	66.6	3.70	14.3	0.0074	*Xpsr116a-3B*
4A	2012	24.7	4.98	28.0	-0.0119	*Xpsr59a-4A*
	2013	24.7	3.19	12.5	-0.0071	*Xpsr59a-4A*
7B	2012	182.5	5.02	28.1	0.0146	*Xglk750-7BL*
Average grain filling rate						
1A	2012	70.8	3.07	18.3	-0.0052	*Xpsr1327b-1A*
2A	2012	94.9	5.01	28.1	-0.0047	*Xpsr919b-2A*
	2013	90.9	4.92	18.6	-0.0031	*Xpsr151-2A*
	2013	126.6	4.76	18.1	-0.0044	*Xglk699b-2AL*
3DL	2012	32.2	3.35	19.8	0.0039	*Xpsr388-3DL*
4A	2012	23.7	6.39	34.3	-0.0061	*Xpsr59a-4A*
4DL	2012	86.0	3.25	19.2	-0.0038	*Xglk302b-4DL*
7B	2012	189.5	5.99	32.6	0.0075	*Xmwg710a-7B*
Maximum grain filling rate						
2A	2012	87.3	4.26	24.4	-0.0187	*Xpsr135-2A*
4A	2012	21.7	3.61	21.1	-0.0203	*Xpsr59a-4A*
7B	2012	192.5	3.53	20.7	0.0249	*Xmwg710a-7B*
Grain filling duration						
5A	2013	208.9	2.93	11.5	-38.3	*Xpsr918b-5A*
**Grain water accumulation**						
**Grain 1**						
Maximum grain water content						
1BS	2013	39.9	3.32	13.0	2.47	*Xgwm18-1BS*
2A	2012	132.6	3.19	18.9	-3.80	*Xglk699b-2AL*
2B	2013	145.8	3.22	12.6	-2.22	*Xglk699a-2BS*
3B	2013	8.1	5.22	19.6	3.27	*Lrk10c-3BS*
4A	2012	20.7	4.70	26.6	-3.84	*Xglk315-4AS*
5A	2012	209.9	4.36	24.9	3.83	*Xpsr918b-5A*
	2013	209.9	9.37	32.4	4.18	*Xpsr918b-5A*
5DL	2012	41.0	4.21	24.2	-6.40	*Xpsr580a-5DL*
	2013	40.0	6.34	23.3	-6.36	*Xpsr580a-5DL*
7B	2012	71.7	3.46	20.3	3.03	*Xglk549-7B*
	2012	193.5	3.88	22.5	4.36	*Xmwg710a-7B*
7D	2013	81.8	3.68	14.3	2.69	*Xpsr662-7D*

Supplementary Table S2. (continued)

Trait/Chromosome	Year	Position (cM)	LOD	R^2	Additive effect	Closest marker
Water absorption rate						
2A	2012	133.6	4.34	24.8	-0.0085	*Xglk699b-2AL*
3A	2013	123.5	3.68	14.3	-0.0071	*Xglk652a-3AL*
3B	2013	10.1	3.16	12.4	0.0063	*Lrk10c-3BS*
4A	2012	49.1	3.92	22.7	-0.0070	*CD16.2-4A*
5A	2012	211.9	5.27	29.3	0.0081	*Xpsr918b-5A*
	2013	210.9	8.42	29.7	0.0094	*Xpsr918b-5A*
5DL	2012	45.0	4.54	25.8	-0.0125	*Xpsr580a-5DL*
	2013	32.0	5.03	19.0	-0.0138	*Xpsr906a-5DL*
6A	2013	2.0	3.27	12.8	-0.0058	*Xpsr008-6A*
7B	2012	68.3	3.29	19.4	0.0059	*Xglk549-7B*
	2012	194.5	3.24	19.2	0.0079	*Xmwg710a-7B*
	2013	67.3	3.14	12.3	0.0057	*Xglk598-7BL*
7D	2012	74.7	3.38	19.9	0.0058	*Xgwm44-7D*
Water loss rate						
1BS	2012	32.8	3.24	19.2	0.0060	*Xpsr634-1BS*
2B	2013	147.8	3.02	11.9	-0.0034	*Xglk699a-2BS*
3B	2013	0.1	4.85	18.4	0.0043	*Xglk683-3BS*
4A	2012	19.7	4.43	25.3	-0.0078	*Xglk315-4AS*
	2013	32.2	3.75	14.5	-0.0043	*Xpsr59a-4A*
5A	2013	210.9	5.31	19.9	0.0049	*Xpsr918b-5A*
5DL	2013	47.0	5.02	18.9	-0.0079	*Xpsr580a-5DL*
7B	2012	193.5	3.26	19.3	0.0085	*Xmwg710a-7B*
7D	2013	82.8	4.13	15.9	0.0044	*Xpsr662-7D*
Time at maximum grain water content						
3A	2013	108.3	3.51	13.7	13.7	*Xglk577-3AL*
6B	2013	55.7	3.12	12.2	-27.4	*Xpsr964-6B*
**Grain 2**						
Maximum grain water content						
2A	2012	94.9	3.21	19.0	-2.47	*Xpsr919b-2A*
	2013	156.0	3.60	14.0	-2.07	*Xpsr630-2A*
2B	2013	145.8	3.16	12.4	-1.90	*Xglk699a-2BS*
3B	2012	80.5	3.49	20.5	2.66	*Xpsr1054-3B*
	2013	8.1	5.50	20.6	2.89	*Lrk10c-3BS*
	2013	80.5	4.86	18.4	2.44	*Xpsr1054-3B*
4A	2012	19.7	7.02	37.0	-3.97	*Xglk315-4AS*
	2013	33.2	3.78	14.6	-2.44	*Xpsr914-4A*
5B	2012	156.2	3.02	18.0	-2.81	*Xpsr580b-5B*
5DL	2013	42.0	3.96	15.3	-4.35	*Xpsr580a-5DL*
7B	2012	71.7	3.26	19.3	2.62	*Xglk549-7B*
	2012	193.5	5.45	30.1	4.47	*Xmwg710a-7B*
	2013	68.3	5.07	19.1	2.59	*Xglk549-7B*
	2013	184.5	4.46	17.0	3.32	*Xglk750-7BL*
7D	2013	83.8	3.29	12.9	2.28	*Xpsr662-7D*

Supplementary Table S2. (continued)

Trait/Chromosome	Year	Position (cM)	LOD	R^2	Additive effect	Closest marker
Water absorption rate						
2A	2012	124.6	3.26	19.3	-0.0069	*Xglk687a-2AL*
	2013	88.3	3.09	12.1	-0.0047	*Xpsr386c-2A*
3B	2013	10.1	3.41	13.3	0.0059	*Lrk10c-3BS*
4A	2012	21.7	4.15	23.9	-0.0061	*Xpsr59a-4A*
5B	2012	159.2	3.49	20.5	-0.0059	*Xpsr580b-5B*
5DL	2013	31.0	3.05	12.0	-0.0098	*Xpsr906a-5DL*
7B	2012	70.7	3.85	22.4	0.0052	*Xglk549-7B*
	2012	193.5	4.91	27.6	0.0081	*Xmwg710a-7B*
	2013	67.3	5.70	21.2	0.0068	*Xglk598-7BL*
	2013	180.5	3.25	12.7	0.0067	*Xglk750-7BL*
Water loss rate						
2A	2013	7.0	4.07	15.7	-0.0037	*Xpsr566c-2A*
3B	2013	6.1	3.39	13.2	0.0038	*Lrk10c-3BS*
	2013	80.5	3.36	13.1	0.0035	*Xpsr1054-3B*
4A	2012	20.7	5.30	29.4	-0.0091	*Xglk315-4AS*
	2013	21.7	7.29	26.3	-0.0058	*Xpsr59a-4A*
6A	2012	78.2	4.27	24.5	0.0101	*Xpsr966-6A*
7B	2012	195.5	3.46	20.4	0.0091	*Xmwg710a-7B*
	2013	190.5	3.74	14.5	0.0053	*Xmwg710a-7B*
Time at maximum grain water content						
3A	2013	112.5	3.84	14.8	15.5	*Xglk577-3AL*
4A	2012	0.1	3.14	18.7	-16.9	*Xglk752-4AS*
**Grain 3**						
Maximum grain water content						
2A	2013	8.9	4.03	15.5	-2.18	*Xpsr566c-2A*
	2013	88.9	4.18	16.1	-2.14	*Xpsr386c-2A*
3B	2013	80.3	3.23	12.6	2.03	*Xpsr1054-3B*
4A	2012	23.7	4.67	26.5	-3.55	*Xpsr59a-4A*
	2013	21.7	5.12	19.3	-2.87	*Xpsr59a-4A*
4DL	2012	95.0	3.07	18.3	-3.28	*Xglk302b-4DL*
7B	2012	190.5	4.45	25.4	4.41	*Xmwg710a-7B*
	2013	65.3	4.67	17.8	2.40	*Xglk598-7BL*
	2013	190.5	4.64	17.6	3.44	*Xmwg710a-7B*
Water absorption rate						
2A	2013	88.9	3.44	13.4	-0.0041	*Xpsr386c-2A*
7B	2013	66.3	5.24	19.7	0.0054	*Xglk598-7BL*
	2013	189.5	3.19	12.5	0.0061	*Xmwg710a-7B*
Water loss rate						
2A	2013	7.0	3.87	15.0	-0.0045	*Xpsr566c-2A*
	2013	117.6	4.18	16.0	-0.0061	*Xglk687a-2AL*
4A	2012	22.7	5.18	28.9	-0.0114	*Xpsr59a-4A*
	2013	21.7	6.54	24.0	-0.0068	*Xpsr59a-4A*
7B	2012	184.5	3.90	22.6	0.0125	*Xmwg710a-7B*
	2013	195.5	4.83	18.3	0.0070	*Xmwg710a-7B*
Time at maximum grain water content						
4A	2012	19.7	3.63	21.2	-34.6	*Xglk315-4AS*

Supplementary Table S2. (continued)

Trait/Chromosome	Year	Position (cM)	LOD	R^2	Additive effect	Closest marker
**Grain dimensions at maturity**						
Grain length						
1DS	2013	0.1	3.0	6.6	0.10	*Xpsr168-1DS*
2A	2012	123.7	3.1	18.2	-0.26	*Xglk687a-2AL*
	2013	143.1	8.6	17.9	-0.16	*PL_AP-2A*
3B	2012	84.5	5.0	28.2	0.26	*Xpsr1054-3B*
	2013	79.3	6.5	13.7	0.15	*Xpsr1054-3B*
4A	2012	23.7	5.8	31.8	-0.27	*Xpsr59a-4A*
5A	2013	110.2	3.1	6.9	-0.11	*Xpsr911-5A*
7B	2012	215.8	3.8	22.1	0.20	*Xglk576-7BL*
Grain width						
2D	2013	99.1	4.3	9.4	-0.04	*Xpsr932-2D*
5A	2013	67.4	3.8	8.3	0.04	*Xglk424-5A*
Grain height						
1BS	2013	31.0	4.4	9.5	0.04	*Xglk317a-1BS*
4A	2012	43.1	4.6	26.1	-0.09	*Xpsr914-4A*
	2013	34.2	5.9	12.5	-0.06	*Xpsr914-4A*
5A	2013	209.9	7.4	15.5	0.07	*Xpsr918b-5A*
5DL	2012	35.0	3.9	22.5	-0.16	*Xpsr580a-5DL*
	2013	37.0	6.9	14.5	-0.12	*Xpsr580a-5DL*
7B	2012	79.6	3.8	22.0	0.09	*Xglk478-7BL*
	2012	184.5	7.6	39.5	0.15	*Xglk750-7BL*
Grain volume						
1A	2012	80.1	3.7	21.6	-2.0	*Xpsr1327b-1A*
1BS	2013	31.0	3.1	6.8	0.8	*Xglk317a-1BS*
2A	2012	122.6	3.2	18.9	-2.7	*Xglk687a-2AL*
	2013	6.0	3.8	8.4	-0.9	*Xpsr566c-2A*
	2013	126.6	6.1	13.1	-1.5	*Xglk699b-2AL*
3B	2012	80.5	3.8	22.0	2.1	*Xpsr1054-3B*
	2013	1.9	3.9	8.4	0.9	*C970a-3B*
	2013	85.5	4.5	9.7	1.1	*Xpsr1054-3B*
4A	2012	21.7	6.6	35.1	-3.0	*Xpsr59a-4A*
	2013	31.2	6.6	14.0	-1.3	*Xpsr59a-4A*
5DL	2013	41.0	4.0	8.7	-1.8	*Xpsr580a-5DL*
7B	2012	78.6	3.8	22.3	2.4	*Xglk478-7BL*
	2012	184.5	7.0	36.7	3.8	*Xglk750-7BL*
Grain length/width						
1DS	2013	1.0	3.7	8.1	0.04	*Xpsr168-1DS*
2A	2013	152.5	6.5	13.7	-0.05	*Xglk278b-2AL*
2B	2013	190.1	3.0	6.6	-0.04	*Xpsr644b-2B*
2D	2013	101.1	5.7	12.1	0.05	*Xpsr540-2D*
3B	2013	54.9	3.1	6.7	0.04	*Xpsr1101b-3B*
5A	2013	68.4	5.5	11.7	-0.06	*Xglk424-5A*
Grain length/height						
3B	2013	61.6	4.5	9.7	0.06	*Xpsr116a-3B*
5A	2012	207.9	3.5	20.4	-0.08	*Xpsr1194-5A*

Supplementary Table S3. Quantitative trait locus (QTL) coincidences between final grain weight and grain filling traits

Chromosome	No. of QTL for Fgw[a]	No. of QTL coincident with those for Fgw																		
		Cs	Igfr	Rgfr	Lgfr	Agfr	Mgfr	Ogf	Gfd	Tmax	Mwc	War	Wlr	Tmwc	Gl	Gw	Gh	Gv	Lw	Lh
2B	1										2		1							
2A.1[b]	2			3		5	2				2	2								
2A.2	2	1	1	2		3	1				2	2	1		2			2	1	
3B.1	2	2		1		2	1				2	2	2					1		
3B.2	3					1					3		1		2			2		
4A	5	2	1	4		3	3	1			5	2	6	2	1		2	2		
5A	2	1	1	1	1	1	1		1		2	2	1				1			1
5DL	2	2	1	1	1	2	1				3	3	1				2	1		
7B.1	2	1		1		2	1				4	5					1	1		
7B.2	3		1	3		4	3				5	4	5		1		1	1		
7D	2										2	1	1							
Total coincident QTL	–	9	5	16	2	23	13	1	1	0	32	23	19	2	6	0	7	10	1	1
Total QTL detected	26	13	5	22	4	31	16	1	2	0	36	26	23	5	8	2	8	13	6	2
Proportion (%)[c]	–	69	100	73	50	74	81	100	50	–	89	88	83	40	75	0	88	77	17	50

[a] Trait abbreviations: Fgw, final grain weight; Cs, carpel size; Igfr, initial grain filling rate; Rgfr, rapid grain filling rate; Lgfr, late grain filling rate; Agfr, average grain filling rate; Mgfr, maximum grain filling rate; Ogf, onset of grain filling; Gfd, grain filling duration; Tmax, the time at maximum grain filling rate; Mwc, grain maximum water content; War, grain water absorption rate; Wlr, grain water loss rate; Tmwc, the time at maximum grain water content; Gl, grain length; Gw, grain width; Gh, grain height; Gv, grain volume; Lw, grain length/width (L/W); Lh, grain length/height (L/H).

[b] Serial number following the chromosome name indicates multiple QTL regions on the same chromosome.

[c] Percentage of the QTL coincident with those for final grain weight to the total QTL number detected for each trait.

Supplementary Table S4. Quantitative trait locus (QTL) coincidences between carpel size and the other grain filling traits

Chromosome	No. of QTL for Cs[a]	No. of QTL coincident with those for Cs																	
		Igfr	Rgfr	Lgfr	Agfr	Mgfr	Ogf	Gfd	Tmax	Mwc	War	Wlr	Tmwc	Gl	Gw	Gh	Gv	Lw	Lh
2A	1	1	2		3	1				2	2	1		2			2	1	
3B	2		1		2	1				2	2	2					1		
4A	2	1	4		3	3	1			5	2	6	2	1		2	2		
5A.1[b]	2														1			1	
5A.2	1	1	1	1	1	1		1		2	2	1				1			1
5B	1																		
5DL	2	1	1	1	2	1				3	3	1				2	1		
6A	1										1								
7B	1		1		2	1				4	5					1	1		
Total coincident QTL	–	4	10	2	13	8	1	1	0	18	17	11	2	3	1	6	7	2	1
Total QTL detected	13	5	22	4	31	16	1	2	0	36	26	23	5	8	2	8	13	6	2
Proportion (%)[c]	–	80	45	50	42	50	100	50	–	50	65	48	40	38	50	75	54	33	50

[a] Trait abbreviations: Cs, carpel size; Igfr, initial grain filling rate; Rgfr, rapid grain filling rate; Lgfr, late grain filling rate; Agfr, average grain filling rate; Mgfr, maximum grain filling rate; Ogf, onset of grain filling; Gfd, grain filling duration; Tmax, the time at maximum grain filling rate; Mwc, grain maximum water content; War, grain water absorption rate; Wlr, grain water loss rate; Tmwc, the time at maximum grain water content; Gl, grain length; Gw, grain width; Gh, grain height; Gv, grain volume; Lw, grain length/width (L/W); Lh, grain length/height (L/H).

[b] Serial number following the chromosome name indicates multiple QTL regions on the same chromosome.

[c] Percentage of the QTL coincident with those for carpel size to the total QTL number detected for each trait.

Supplementary Table S5. Quantitative trait locus (QTL) number detected for Grain 1, 2 and 3

Grain	Fgw[a]	Cs	Igfr	Rgfr	Lgfr	Agfr	Mgfr	Ogf	Gfd	Tmax	Mwc	War	Wlr	Tmwc
Grain 1	11	7	5	8	3	12	7	0	0	0	12	13	9	2
Grain 2	8	4	0	6	1	11	6	1	1	0	15	10	8	2
Grain 3	7	2	0	8	0	8	3	0	1	0	9	3	6	1
Total	26	13	5	22	4	31	16	1	2	0	36	26	23	5

[a] Trait abbreviations: Fgw, final grain weight; Cs, carpel size; Igfr, initial grain filling rate; Rgfr, rapid grain filling rate; Lgfr, late grain filling rate; Agfr, average grain filling rate; Mgfr, maximum grain filling rate; Ogf, onset of grain filling; Gfd, grain filling duration; Tmax, the time at maximum grain filling rate; Mwc, grain maximum water content; War, grain water absorption rate; Wlr, grain water loss rate; Tmwc, the time at maximum grain water content.

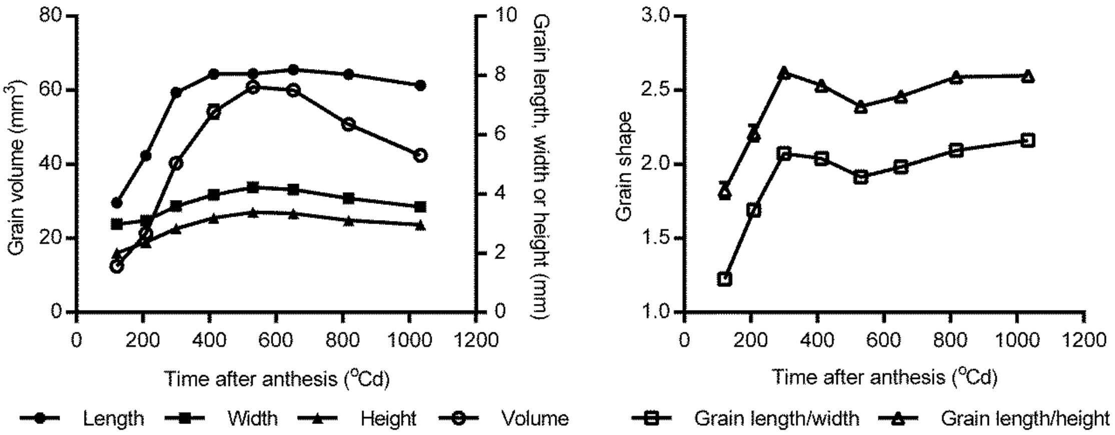

Supplementary Fig. S1. Grain expansion dynamics in 2013. Bars depict the standard errors of the means (SEM).

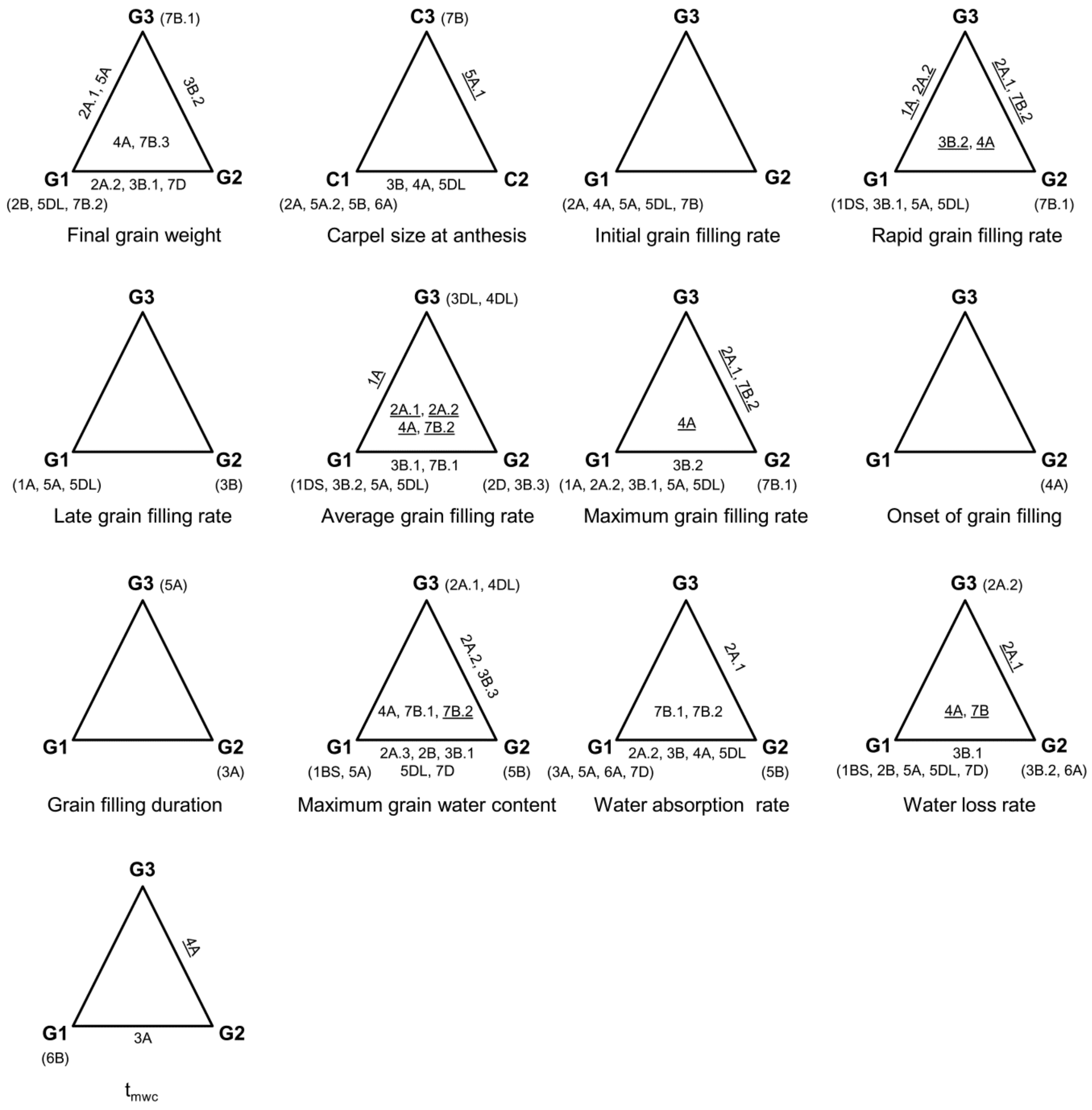

Supplementary Fig. S2. Quantitative trait locus (QTL) comparisons between different grains within spikelets. C1 (G1), C2 (G2) and C3 (G3) represent Carpel 1 (Grain 1), Carpel 2 (Grain 2), and Carpel 3 (Grain 3), respectively. Chromosome names following G1 (C1), G2 (C2), and G3 (C3) in the parentheses show the locations of position-specific QTL. Serial numbers after chromosome names indicate multiple QTL detected on the same chromosomes for a trait. The shared QTL with higher additive effects in G3 (C3) are marked by the underlines. The QTL shared by any two of the grains are placed at the middles outside the triangles, while these shared by all the three grains are placed inside the triangles. Trait abbreviation: t_{mwc}, the time at maximum grain water content.
